# Photocurable Thiol–Ene/Nanocellulose Elastomeric Composites for Bioinspired and Fluorine-Free Superhydrophobic Surfaces

**DOI:** 10.1021/acsami.4c16445

**Published:** 2024-10-24

**Authors:** Alper Balkan, Enrico Sola, Feyza Karasu, Yves Leterrier

**Affiliations:** Laboratory for Processing of Advanced Composites (LPAC), École Polytechnique Fédérale de Lausanne (EPFL), CH-1015 Lausanne, Switzerland

**Keywords:** thiol−ene photopolymerization, cellulose nanofibrils, polymer nanocomposites, UV nanoimprint lithography, superhydrophobic surfaces

## Abstract

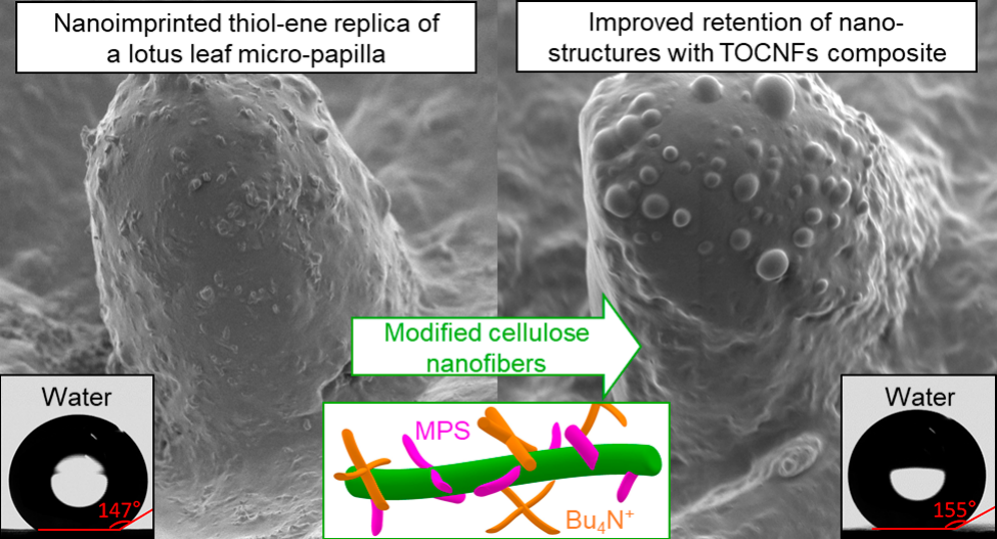

Artificially prepared superhydrophobic surfaces toward a self-cleaning “lotus effect” and anticontamination performance have become critically important in the past few years. However, most approaches to create the required topology with a hierarchical roughness comprise several manufacturing steps of varying practicality. Moreover, the desired low surface energy is in most cases achieved with fluorinated moieties that are currently criticized due to biological and environmental hazards. In this work, rapidly photocuring but weak thiol–ene resins were reinforced with cellulose nanofibrils (CNFs) to replicate lotus leaves via one-step UV nanoimprint lithography. The CNFs were surface-modified using countercation exchange of carboxyl groups and grafting of thiol and methacrylate functionalities. The formulation methodology resulted in free-flowing, shear-thinning composite resins without surfactants or dispersants. The rheological and photo-cross-linking behavior of the resins, the thermal stability, the mechanical performance, and the hydrophobicity of the cured composites were characterized. Notably, the surface modifications increased the as received fibril diameter (1.9 ± 0.6 nm) by 1.6–2.3 nm and raised the fibril–resin compatibility. The resins underwent rapid polymerization and the high thermal stability of thiol–enes was retained. The methacrylated nanofibrils (10 vol %) significantly strengthened the rubbery network, outperforming the neat thiol–ene polymer in terms of hardness (3.4×), reduced modulus (5.8×), and wear resistance (>100×). Moreover, the surface of lotus-texturized composites was superhydrophobic with a water contact angle of 155°, higher than that of the neat polymer (147°), and was self-cleaning. These CNF composite resins are compatible with fast-cure processes such as 3D printing and roll-to-roll processing, are exempt of fluorine or any other hydrophobization treatment, and are extremely wear-resistant.

## 1 Introduction

Synthetic superhydrophobic surfaces have been developed for several decades in academia and industry for their characteristics such as anticontamination, self-cleaning, and antisticking in a variety of areas including automotive, ship building, and textiles.^1^ The best example of a natural self-cleaning surface is that of the lotus leaf, hence the name Lotus Effect,^2^ which combines a double- or multistructured micro/nano-roughness^3^ and a low surface energy. Hierarchical roughness can be created using top-down (e.g., templation, plasma treatment) and bottom-up (e.g., chemical deposition, colloidal assemblies) processes or their combinations,^1,4^ whereas low energy surfaces are often based on treatments with fluoropolymers.^1,5^ These substances have been lately receiving criticism due to associated environmental and biological hazards,^6^ requiring the development of alternative, more environmentally benign material and manufacturing strategies. In fact, a fundamental question is whether a surface with hierarchical roughness can be superhydrophobic without low surface energy treatment.

Focus of the present work to replicate the micro/nano-patterned surface of lotus leaves is photopolymerization via thiol–ene chemistry. Photopolymerization of organic precursors brings together a set of advantages such as rapid drying, solventless formulations and reduced energy consumption, and its relevance for biobased materials is rapidly growing.^7^ The radical photopolymerization achieved with thiol–ene chemistry enables rapid, oxygen insensitive formation of homogeneous polymer networks devoid of side products^8^ and with minimal shrinkage, warpage, and internal stress in contrast to more conventional monomers, e.g., methacrylates.^9^ These attributes proved to be beneficial for the preparation of tailored polymer networks targeting specific technical applications such as additive manufacturing^10^ and nanoimprinting.^11^ In the past years there have been attempts at making superhydrophobic surfaces via thiol–ene chemistry as well, yet again mostly involving fluoropolymers.^12^

One critical challenge of thiol–ene networks is their poor mechanical performance arising from the formation of flexible thio–ether linkages, which limit their application in some fields where more robustness is desired.^8^ To overcome this limitation, an efficient solution is the incorporation of rigid fillers to reinforce the network structure. In particular, nanofillers have the potential to enhance the mechanical properties of composite materials at rather low concentrations (<10 wt %)^13^ as well as minimally interfere with their optical transmittance through the control of particle size.^14^ Over the years, nanocomposites based on various forms of cellulose have been applied in numerous areas including coating and packaging, hydrogels, automotives, electronics,^15^ and additive manufacturing.^7^ CNFs are attractive candidate biomaterials for polymer composites owing to their superior characteristics compared to organic resins such as biocompatibility, biodegradability, and process-induced anisotropy of the composite due to high aspect ratio, tunable surface chemistry, and reinforcement capability. Moreover, this class of materials can spearhead the efforts of replacing fossil oil-based products with biodegradable^16^ and/or biobased ones.^17^ However, despite the potential benefits, widespread use of CNFs in photopolymerization, in particular with thiol–ene systems, is yet to be reached, following previous studies with cellulose nanocrystals (CNCs).^7,18^ In fact, CNFs have considerably high tribological performance,^19^ high strength and stiffness,^15^ and can act as stronger mechanical reinforcers for polymers than CNCs.^20^ Additionally, networks formed by interacting CNFs can effectively bridge crazes due to fibril interlocking and fibril pullouts, which can lead to elevated values of failure strain and tensile strength.^15^ The introduction of negatively charged groups, such as carboxyl or carboxymethyl groups, on CNFs leads to an improvement in the delamination of the nanofibrils, thanks to electrostatic repulsion between the negatively charged CNFs.^21^ A common example is CNFs oxidized by 2,2,6,6-tetramethylpiperidine-*N*-oxyl (TEMPO) mediation (TOCNFs). This chemo-mechanical production strategy of TOCNFs, which is reported to be environmentally friendlier compared to methods used for other cellulose nanomaterials like CNCs,^22^ leads to isolated nanofibrils of 3–10 nm in diameter with lengths reaching a few micrometers.^22,23^ Water dispersions of TOCNFs are almost fully transparent;^24^ however, achieving a similar level of dispersion in an organic medium is nontrivial and relies on surface modifications.^7,25,26^ Modifiers like quaternary ammonium salts were proven effective for ionic compatibilization of nanocellulose materials.^27^ Furthermore, changing the counterion of the carboxylate is known to affect material properties such as thermal stability and even the true density of the single fiber.^28^ In recent years, there have been multiple successful efforts at preparing superhydrophobic films and foams based on fluorinated as well as nonfluorinated nanocellulose.^29,30^ Nevertheless, it is highly common to encounter multistep manufacturing, sometimes involving time-consuming, cost-inefficient methods such as chemical vapor deposition or freeze-drying. Moreover, conventional spraying requires low viscosity resins, causing solvent evaporation during the manufacturing. Therefore, a resin strategy compatible with facile and scalable manufacturing methods requiring higher constant viscosities is still lacking.

The UV nanoimprint lithography (UVNIL) process was selected for the present work for its simplicity, cost-effectiveness, and high throughput.^31^ UVNIL offers sub-100 nm resolution^32^ and is fully compatible with up-scaling via, e.g., roll-to-roll manufacturing.^33^ This technique was successfully used to produce micro- and nanostructured surfaces^34^ that are superhydrophobic or superhydrophilic,^5^ including rose petals^35,36^ and lotus leaves.^37^ Also notice that thiol–ene polymers formed by UVNIL can host fewer defects than common photopolymers due to their oxygen tolerance^31^ and are gaining attention as photoresists owing to their thermal endurance and etch resistance.^38,39^

In this work, a methodology was introduced for reinforcing thiol–ene resins with different surface-modified TOCNFs. Tetrabutylammonium was used as a hydrophobic carboxylate countercation in combination with thiol or methacrylate surface functionalities, which were studied for morphological changes in fibrils, additional cross-linking, and reduced polarity benefits. The flow behavior and photo-cross-linking of the resin formulations were monitored, and the thermal stability and mechanical performance of the cured materials were analyzed. The hydrophobicity of texturized surfaces replicated from a lotus leaf without the application of a conventional hydrophobization treatment was also investigated.

## 2 Materials and Methods

### 2.1 Materials

The multifunctional thiol compound, trimethylolpropane tris(3-mercaptopropionate) (TMPTMP, ≥95%, Sigma-Aldrich), monomer trimethylolpropane diallyl ether (TMPDE, 90%, Sigma-Aldrich), photoinitiator 2,2-dimethoxy-2-phenylacetophenone (DMPA, >98%, TCI Chemicals), and radical scavenger butylated hydroxytoluene (BHT, Sigma-Aldrich) were used as received. The TOCNFs were supplied in the form of a water suspension (∼1 wt %) by the University of Maine, and the chemicals for surface modification comprising 3-(methacryloyloxy) propyltrimethoxysilane (MPS, 98%, Sigma-Aldrich), 3-(mercaptopropyl) trimethoxysilane (MPTMS, 95%, Sigma-Aldrich), and tetrabutylammonium hydroxide (Bu_4_NOH, 40 wt % in water, Sigma-Aldrich) were purchased and processed as described in the following. The structures of all the mentioned chemicals are shown in Figure S1.

### 2.2 Surface Modifications on TOCNFs

The TOCNFs surface was modified using Bu_4_NOH and hydrolyzed silanes, following the works of Shimizu et al.,^27^ Lu et al.,^40^ and Galland et al.^41^ The surface modifiers Bu_4_NOH, MPS, and MPTMS were added as a 1:1 weight ratio or more with respect to the calculated TOCNFs weight present in the as received water suspension to ensure the excess of modifiers. Briefly, the TOCNFs compatibility with hydrophobic media was amplified by Bu_4_NOH, which was used to replace the transient protons of the TOCNFs carboxyl groups as well as the sodium cations at the TOCNFs carboxylates with the bulky tetrabutylammonium cations leading to a quaternary alkylammonium carboxylate salt.^27^ The cation exchange process was initiated by the mixing of 6 mL of as received TOCNFs water suspension (60 mg of TOCNFs) with 175 mg of 40 wt % Bu_4_NOH–water solution (70 mg of Bu_4_NOH), followed by ultrasonication (Digital Sonifier 550, Branson Ultrasonics Corporation, USA) for 15 min with a duty cycle of 15 s pulses (55 W) and 30 s of pause for mixing at room temperature. In the next step, the suspension was solvent exchanged with acetone via thrice repeated ultracentrifugation at 6000 rpm (Heraeus Biofuge Promo Centrifuge, Thermo Scientific, Switzerland), supernatant removal, and addition of fresh acetone. The solvent exchange step simultaneously removed the unbound Bu_4_NOH.

The hydrolyzed silanes with the methacrylate (MPS) and thiol (MPTMS) moieties were chemically attached to the TOCNFs surface by condensation with the hydroxyl groups. Prehydrolyzed MPS (hyMPS) was prepared by mixing 10 g of MPS with 2.2 g of pH 1 water (consisting of ∼0.13 g of hydrochloric acid and ∼2.07 g of Milli-DI water) and 1.25 g of ethanol, resulting in a solution of pH 4. The solution was hydrolyzed overnight at room temperature. Water and ethanol, which were initially added and also generated during hydrolysis, were evaporated at 70 mbar and 50 °C for 1.5 h. The same procedure was used to prepare prehydrolyzed MPTMS (hyMPTMS). The molecular structures of hyMPS and hyMPTMS are shown in Figures S2 and S3. Changes in the silane chemical structures due to hydrolysis are shown in Figure S4. The modification of TOCNFs (60 mg) with hyMPS (70 mg) and hyMPTMS (70 mg) was done following the solvent exchange step by ultrasonication assistance with the aforementioned parameters. Subsequently, the formulation underwent repeated solvent washing with acetone by a combination of centrifugation at 6000 rpm, removal of supernatant, and addition of fresh acetone to fully remove the unbound as well as the physically adsorbed silanes hyMPS/hyMPTMS on cellulose.^41^ The washing was concluded with another ultrasonication step operating with a duty cycle of 15 s pulses (165 W) and 1 min pauses for 75 min to ensure adequate dispersion.

### 2.3 Preparation of Thiol–Ene Resins with Surface-Modified TOCNFs

**Figure 1:**
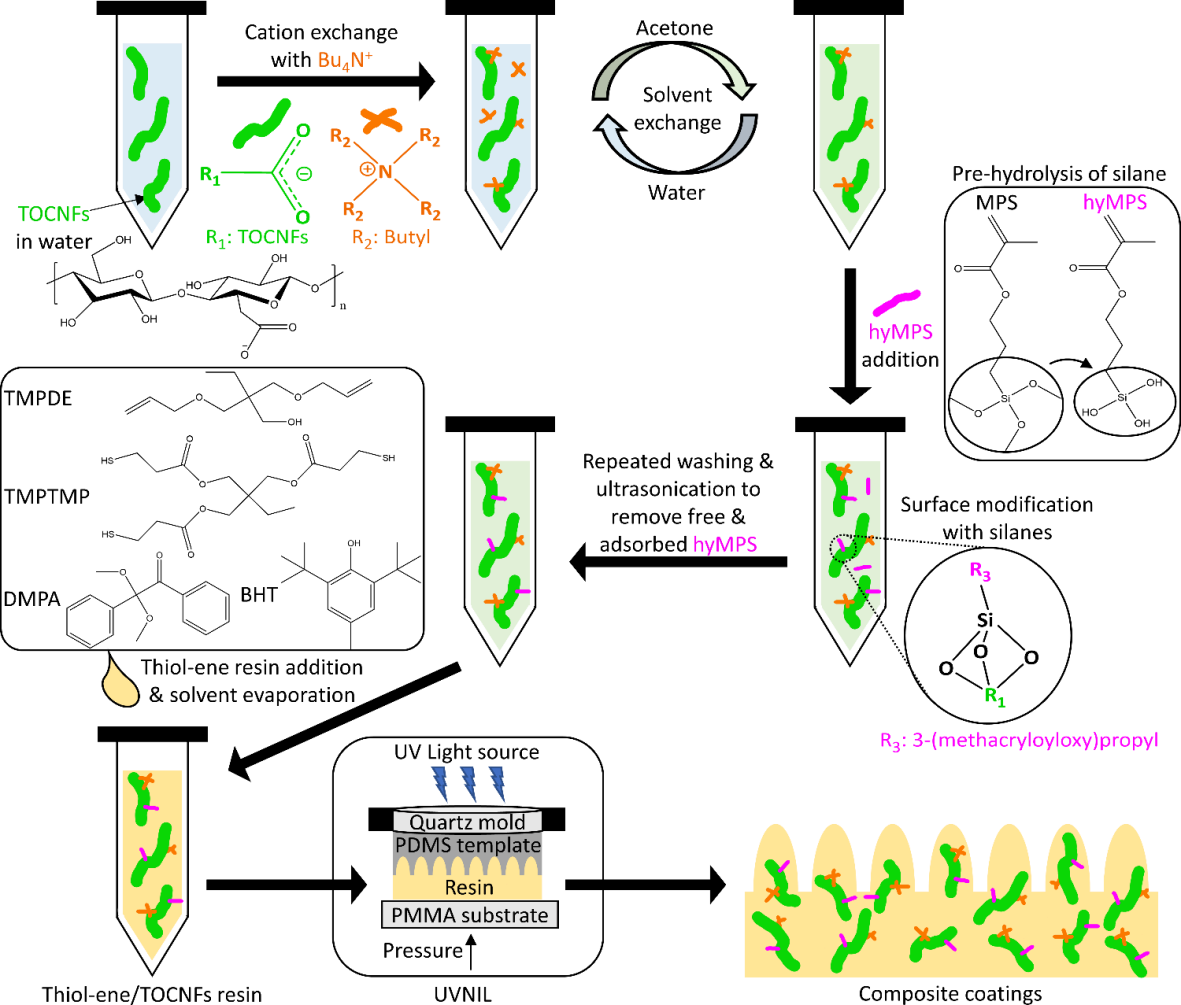
Sketch of the process steps to prepare UV-cured thiol–ene composites with Bu_4_NOH-hyMPS-TOCNFs. The same steps were used to prepare the composites with Bu_4_NOH-hyMPTMS-TOCNFs.

The thiol–ene mixture preparation began by mixing calculated amounts of TMPDE and TMPTMP in a separate tube to obtain a thiol–ene molar functionality ratio of 1:1 as well as to achieve the targeted volume of photocurable liquid phase ensuring the estimated volume concentration of TOCNFs inside the resin. The photoinitiator DMPA (1 wt %) and the radical scavenger BHT (9 mmol/L dose based on the work of Esfandiari et al.^42^) were added to the thiol–ene formulation and mixed for ∼2 min at room temperature until homogeneity was reached. This photocurable mixture, which served as reference resin, was then poured into the first tube containing the unmodified or modified TOCNFs and subjected to a final ultrasonication step with the same parameters as for the TOCNFs modification with the prehydrolyzed silanes. The remaining acetone was evaporated overnight at room temperature. A silicon oil bath set to 50 °C was used to aid the evaporation of acetone in samples requiring longer than overnight drying. The complete solvent evaporation was verified by reaching a constant weight on a milligram-sensitive scale. The volume fraction of TOCNFs was calculated from its measured weight fraction using density values of 1.46 and 1.3 for TOCNFs^23^ and tetrabutylammonium-modified TOCNFs,^28^ respectively. The density of the silane-functionalized TOCNFs was assumed to be equal to that of the tetrabutylammonium-modified TOCNFs. Different concentrations of TOCNFs were produced depending on the type of modifications. In the case of unmodified and tetrabutylammonium-modified TOCNFs, a single concentration of 10 vol % was produced. For the resins with double-modified nanofibers, namely Bu_4_NOH-hyMPS-TOCNFs and Bu_4_NOH-hyMPTMS-TOCNFs, suspensions with loadings of 0.5 and 1 vol % were prepared, the hyMPS version additionally including concentrations of 5 and 10 vol %. A schematic depicting the preparation and UVNIL of one of the composite resin types is displayed in Figure 1.

### 2.4 Photocuring of Thiol–Ene/TOCNFs Composite Films

Prior to UV curing, the composite resins were freshly ultrasonicated and coated on a glass substrate (ISO 8037/1 glass slides, 76 × 26 × 1 mm^3^) using three different techniques. Several micrometer thick coatings were drop-cast on glass slides placed inside a cavity with the area of a glass slide located in a polydimethylsiloxane (PDMS, Sylgard 184 Silicone Elastomer Kit, Dow, Germany) mold to avoid spilling of the resin from the glass slide during solvent evaporation. In addition, resins, whose solvent content following preparation could rapidly evaporate unhindered by the resin, were spin-coated at 300 rpm for 30 s followed by a second step lasting 30 s with rotation speeds ranging from 500 to 3000 rpm. Third, approximately 200 μm coatings were produced using a doctor blade for water contact angle and UV–visible light (UV–vis) spectroscopy measurements. The composite coatings were cured using a high-pressure Hg UV flood curing lamp (ECE PC-2000, Dymax, USA) for 1 min at ∼110 mW/cm^2^ irradiance, as measured using a Silver Line UV radiometer (CON-TROL-CURE, Germany).

### 2.5 UVNIL Replication of Superhydrophobic Micro- and Nanostructured Lotus Patterns

The surface of the composite coatings was texturized via a UVNIL replication of *Nelumbo lutea* (lotus, harvested on July 19th of 2022) leaves. The freshly obtained leaves were used within a few hours to create negative PDMS templates by cutting and taping the leaves onto Petri dishes in the form of 2 × 2 cm^2^ squares. Then, PDMS precursor (Sylgard 184) mixed with hardener (10:1 ratio) was poured into the Petri dish until the leaves were covered in excess and air bubbles were removed for 20 min via a vacuum oven. After a curing time of 48 h at room temperature, PDMS squares of 2 × 2 cm^2^ area were cut to be used as negative templates to replicate the micro- and nanostructured surface of the lotus leaves in the composite resin by the UVNIL process. To initiate the procedure, a photocurable thiol–ene resin with or without the TOCNFs was deposited evenly onto a 1 mm thick poly(methyl methacrylate) substrate by doctor blading. The micro- and nanostructured side of the previously prepared PDMS negative template was positioned in contact with the resin, and the assembly was placed within a custom-built UVNIL setup. A constant pressure of 2 bar was applied to the poly(methyl methacrylate) substrate from below for 2–5 min, thereby allowing the resin to infiltrate the micro- and nanostructured details of the negative template. UV light with an intensity ∼110 mW/cm^2^ was applied over the flat side of the PDMS template (Figure 1) for 3 min while holding the pressure constant. Lastly, the PDMS negative template was gently removed to obtain the nanostructured thiol–ene film.

### 2.6 Characterization of Resins and Cured Materials

All of the characterization methods, namely, infrared spectroscopy, zeta potential, UV–vis, thermogravimetric analysis, (photo)rheometry, photodifferential scanning calorimetry, nanoindentation tests, wear tests, laser scanning confocal microscopy, atomic force microscopy, scanning electron microscopy, water contact angle measurements, and self-cleaning tests, are detailed in the Supporting Information file. Notice that different light intensities were used in the photohyphenated methods in order to maximize their resolution. The resulting different conversion kinetics were taken into account through the energy dose equivalence, in which the same conversion is achieved when the product of the exposure time and light intensity is constant.^43^

## 3 Results and Discussion

### 3.1 Surface Modification of TOCNFs

The suspension of unmodified TOCNFs in water was transparent, whereas in acetone or in the thiol–ene resin these led to opaque white suspensions or even sedimented, indicating the poor dispersion quality regardless of the chosen dispersion process. High-shear mixing up to 25,000 rpm (Ultra-Turrax T25, IKA-Werke GmbH & Co. KG, Germany) as well as ultrasonication at 275 W were attempted without success. The zeta potential of the Bu_4_NOH-modified TOCNFs in water was found to be lower than that of the unmodified TOCNFs, an indication of the reduced stability of the modified nanofibrils in aqueous medium (Table S1). In contrast, suspensions of the double-modified TOCNFs (Bu_4_NOH and hyMPS/hyMPTMS) in the thiol–ene resin exhibited increased transparency, which is a qualitative indication of an improved dispersion quality. However, it is worth mentioning that increasing the concentration up to 10 vol % resulted in a resin with reduced transparency, as shown in Figure S5, which could also be exacerbated following the photocuring by the surface roughness of the formed film (Figures S6–S8).

**Figure 2:**
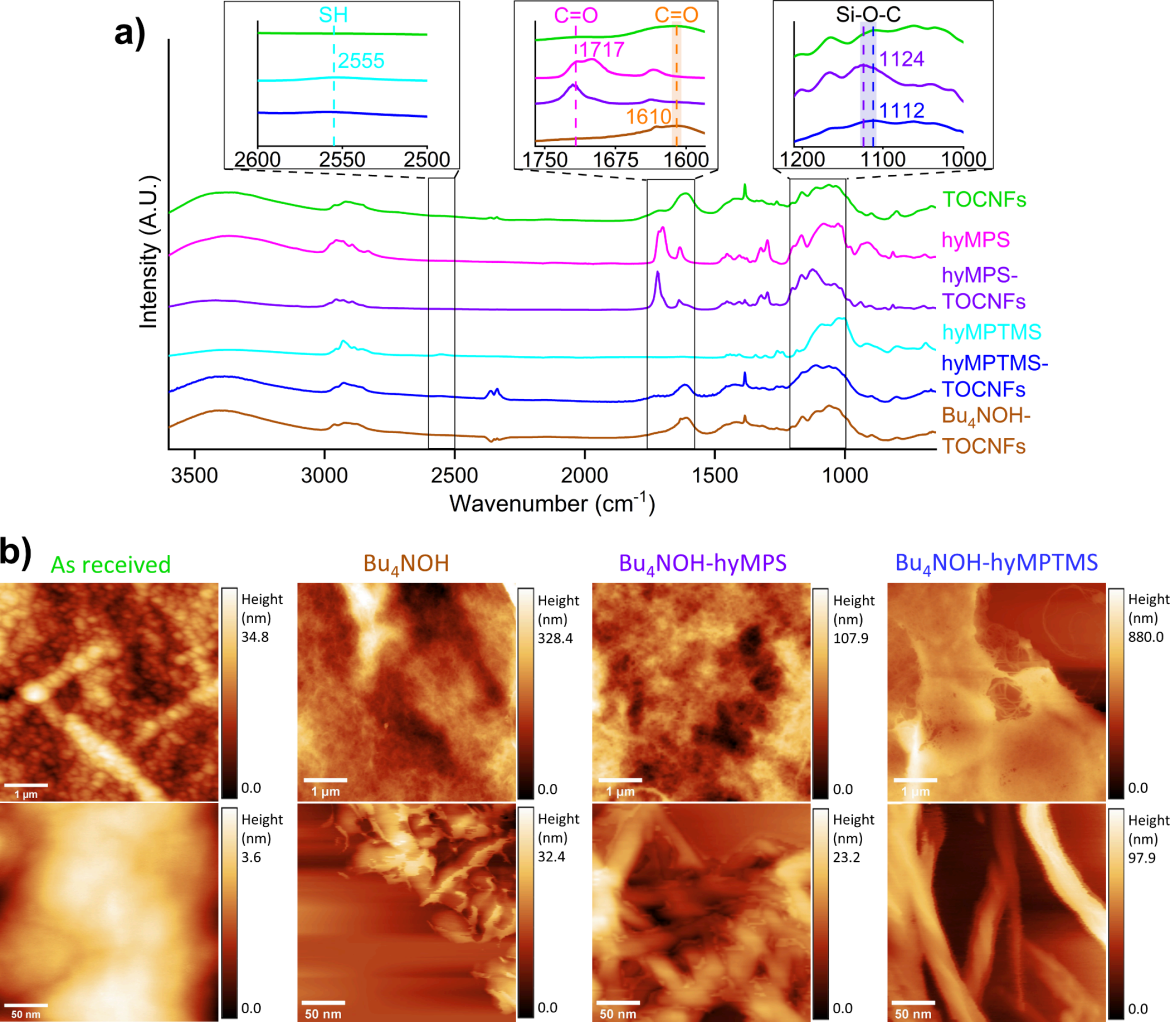
(a) FT-IR spectra of dried TOCNFs (as received), prehydrolyzed MPS, dried MPS-modified TOCNFs, prehydrolyzed MPTMS, dried MPTMS-modified TOCNFs, and dried Bu_4_NOH-modified TOCNFs. (b) AFM images of as received, Bu_4_NOH-modified, Bu_4_NOH-MPS double-modified, and Bu_4_NOH-MPTMS double-modified TOCNFs dried on mica.

Figure 2a shows the FT-IR spectra of the unmodified and modified TOCNFs. The presence of chemically grafted hyMPS on the cellulose surface was evidenced with the relative increase of the carbonyl group peak at 1717 cm^–1^ that was already present in the spectrum of cellulose nanofibrils due to TEMPO-mediated oxidation. The grafting of hyMPTMS was revealed with the appearance of a thiol peak (2555 cm^–1^)^9^ with low amplitude. The presence of tetrabutylammonium bound to the surface of cellulose nanofibrils was demonstrated by the shift of the C=O carbonyl group peak from 1717 to ∼1610 cm^–1^ belonging to TOCNFs carboxyl groups due to the formation of the stronger ionic interaction between COO^–^ and Bu_4_N^+^ leading to decreased electronic density at the carboxyl group. The expected peak at ∼1610 cm^–1^ however was nontrivial to show explicitly as the same region of the as received TOCNFs already hosted a carboxylate peak stemming from the sodium cations.^44^ In addition, the scissoring vibration of adsorbed water at the 1632–1638 cm^–1^ region could be seen on multiple spectra. Moreover, the hyMPTMS peaks of Si–O–Si formation at 1035 cm^–1^ and O–H at 3400 cm^–1^, which was less than hyMPS, indicated that these silanes underwent an extent of condensation,^45^ which presumably occurred during the prehydrolysis. Furthermore, acquiring quantitative information about the Si–O–C vibrational mode between the silanes and the TOCNFs was challenging due to the readily absorbing C–O related modes of TOCNFs in the corresponding region^40^ as well as the potentially overlapping Si–O–Si vibrational modes.^46^ Nevertheless, the silane-modified TOCNFs displayed their strongest peaks at 1112 and 1124 cm^–1^, following hyMPTMS and hyMPS treatments, respectively. This new region, without excluding partial Si–O–Si influence, was ascribed to Si–O–C formation^46^ due to the weaker absorption of the as received TOCNFs as well as the surface modifiers hyMPTMS and hyMPS themselves in that region. A complementary chemical analysis of surface modifiers and cellulose is possible, although it can be nontrivial and lack high quantitative accuracy, through solid-state nuclear magnetic resonance spectroscopy^47^ or dynamic vapor sorption with deuterium exchange.^48^

To monitor the modification-induced morphological variations among the TOCNFs, atomic force microscopy was utilized on fibrils dried on mica, as shown in Figure 2b. The as received TOCNFs appeared heavily intertwined to the extent that the fibril bundles could reach up to several hundreds of nanometers in width. Moreover, spherical formations were detected along the bundles, whose presence could be possible again due to the entangled fibrils. The individual TOCNFs were described by their heights, whose average was 1.9 ± 0.6 nm (Figures S9–S16), in good agreement with the previously reported height values of TOCNFs scanned with AFM (1.7 ± 0.6 and 1.6 ± 0.6 nm as in ref (49) and ∼2.0 nm as in ref (50)). Notice that these values are lower than the generally reported TOCNFs diameters of 3–4 nm (commonly observed with transmission electron microscopy).^23^ Jakubek et al.^51^ analyzed sizes of CNCs with AFM, TEM, and X-ray diffraction and ascribed this AFM–TEM difference to reasons such as the lateral association of nanocellulose and difficult-to-define particle edges in TEM as well as the compression of particles by the tip in AFM. Fibril widths in images were not used for individual fibril size determination in this work to avoid tip broadening effects on the *x* and *y* axes.^49^ The modified TOCNF varieties prepared in this study, namely, Bu_4_NOH-TOCNFs, Bu_4_NOH-hyMPS-TOCNFs, and Bu_4_NOH-hyMPTMS-TOCNFs, did not exhibit similar severely intertwined, bulkier morphologies evidenced with the as received TOCNFs. Moreover, the single fibrils of the three modified TOCNF varieties were on average thicker than the as received TOCNFs. The double-modified Bu_4_NOH-hyMPS-TOCNFs (3.9 ± 1.5 nm) and Bu_4_NOH-hyMPTMS-TOCNFs (4.2 ± 1.5 nm) were slightly thicker than the single-modified Bu_4_NOH-TOCNFs (3.5 ± 1.3 nm). Besides, the length of the individual fibrils was found to remain in the same multiple micrometer range upon chemical modification, within reported TOCNFs lengths.^22^ Thus, the surface modifications increased the fibril diameter and reduced the fibril–fibril interactions.

### 3.2 Flow Behavior of the Thiol–Ene Composite Formulations

**Figure 3:**
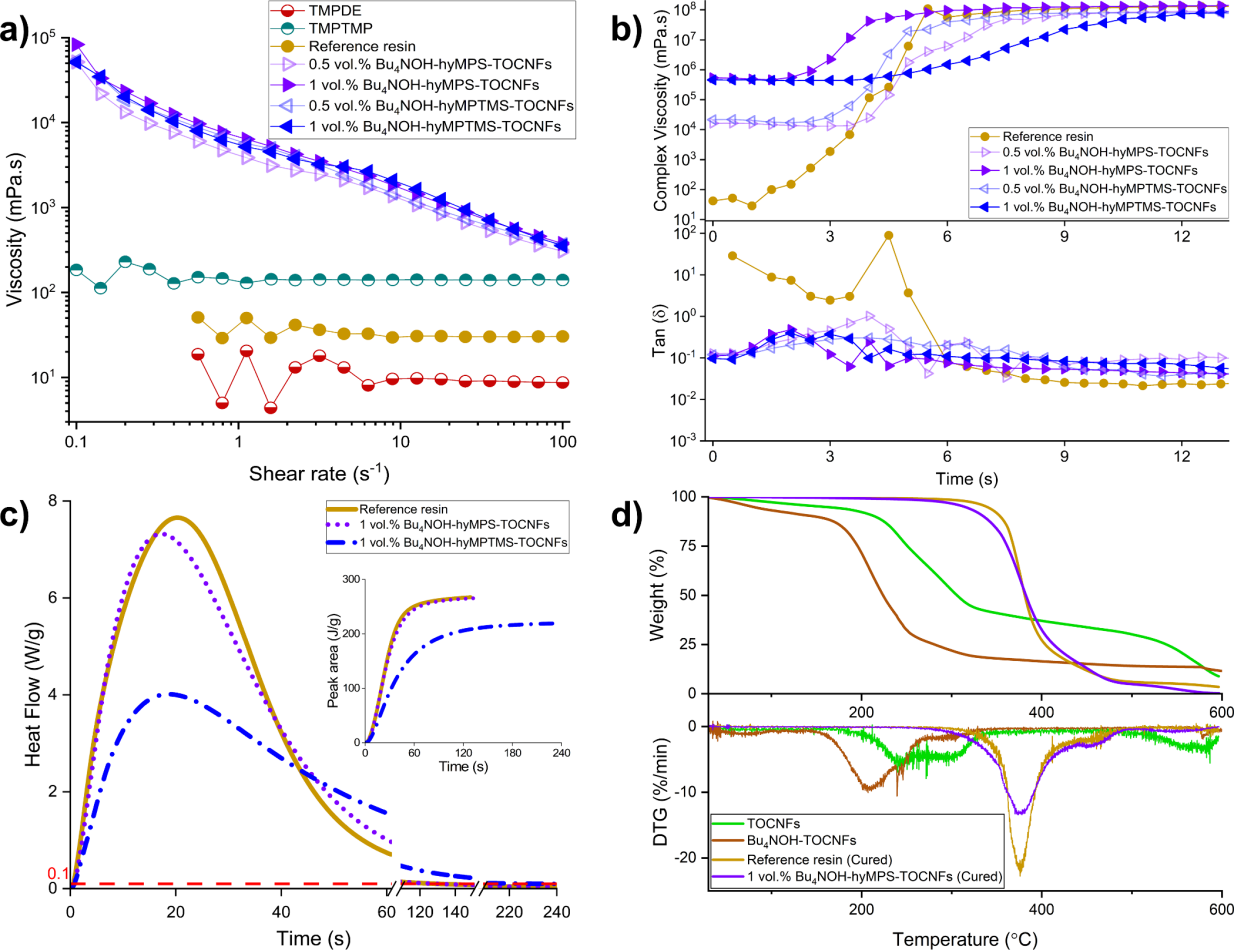
(a) Flow curves of TMPDE and TMPTMP monomers and reference and nanocomposite resins before the UV-curing step (oscillating values were due to lower torque limit). (b) Photorheometry curves of reference and nanocomposite resins, subjected to ∼15 mW/cm^2^ light intensity at time *t* = 0 s to analyze the photo-cross-linking kinetics. (c) Heat flow and peak area curves of reference and nanocomposite resins with 1 vol % loading of Bu_4_NOH-hyMPS-TOCNFs and Bu_4_NOH-hyMPTMS-TOCNFs, subjected to 0.5 mW/cm^2^ light intensity. (d) TGA and derivative TGA curves of dried TOCNFs and Bu_4_NOH-TOCNFs powders and UV-cured reference nanocomposite resins with 1 vol % of Bu_4_NOH-hyMPS-TOCNFs tested in air with a constant ramp rate of 10 °C/min.

Figure 3a shows the flow curves of the monomer precursors and TOCNFs suspensions. The precursors and thiol–ene resin were Newtonian, with TMPTMP and the thiol–ene resin being approximately 14 times and 3 times more viscous that TMPDE, respectively. The suspensions with surface-modified TOCNFs were far more viscous due to the improved dispersion state of the nanocellulose^52^ and were shear-thinning with power-law exponents of 0.36 ± 0.02 and 0.31 ± 0.02 for 0.5 and 1 vol % loaded suspensions, respectively. At higher nanofiber loadings (5 and 10 vol %), the solvent evaporation became unachievable within a reasonable time scale, and only suspensions with loadings up to 1 vol % were analyzed for flow behavior to elude the solvent effect. In fact, when varying the concentration of TOCNFs from 0.5 to 1 vol %, the increase in viscosity was rather low, presumably due to reduced dispersion quality, correlated with reduced transparency (Figure S8). Furthermore, the difference in the type of modifications did not cause a significant change in viscosity.

### 3.3 Photocuring of Thiol–Ene Resins with Modified TOCNFs

The photocuring of the reference resin was checked by comparing its FT-IR spectrum with that of the two monomers, and the full conversion was approved by the disappearance of C=C double bonds of allyl ether and −SH bonds of thiol compounds (Figure S17). The photocuring dynamics of the composite suspensions was analyzed by means of photorheology, and the results are shown in Figure 3b. It was observed that all formulations undergo rapid photo-cross-linking on the order of seconds, eventually reaching a plateau viscosity (upper torque limit). The tan(δ) values of all nanocomposite suspensions were below 1 before curing, which implies that these were gel-type fluids. The variation in curing profiles correlated with the TOCNFs presence and concentration as well as the chosen surface modifier type. First, a delay was present in all composite resins before an onset of complex viscosity increase was observed, in contrast with the reference resin, whose viscosity immediately increased upon light exposure. This delay was associated with the presence and interactions of nanofibers among themselves as well as the surrounding liquid phase of resin leading to a necessary level of cross-linking within the liquid phase before any noticeable complex viscosity increase was possible. Therefore, the initially constant complex viscosities of the composite resins were in stark contrast with their tan(δ) values showing increase upon light exposure. Moreover, the onsets of complex viscosity increase correlated with the approximate peak locations of their respective tan(δ) profiles pointing toward the required structural change in claim. Second, the slopes of the complex viscosity curves during the initial stage of cross-linking differed between the double-modified hyMPS and hyMPTMS TOCNFs. At a concentration of 1 vol %, the suspensions with hyMPS-TOCNFs reacted faster than the resin, whereas the opposite behavior was observed with the hyMPTMS-TOCNFs. The slower photocuring of the resin with hyMPTMS-TOCNFs could be partially attributed to the lower thiol–ene reaction rate associated with alkyl thiols (thiol end of the silane) when compared with propionate thiols (TMPTMP).^53,54^ The sulfur–hydrogen bond in propionate thiols is reported to be weaker, which is caused by the hydrogen bonding between the thiol and the ester carbonyl,^53^ making the propionate thiols better chain transfer agents.^54^ The thiol silane grafted on TOCNFs, however, is less likely to be affected by the mentioned hydrogen bond effect due to the steric hindrance of fibrils even though the resin contains mobile propionate thiols. Third, the variation of complex viscosity slopes was more pronounced with the 1 vol % loaded samples compared to the 0.5 vol % loaded counterparts, which had very similar if not intertwining profiles to both the reference resin and each other. Thus, the concentration of TOCNFs in these resins showed the strongest influence on the measured viscosities. These results were reproducible, as shown in Figure S18, and they highlight the complex influences of the surface chemistry and concentration of TOCNFs on photopolymerization kinetics. In fact, the allyl–ether TMPDE with higher thiol–ene reactivity compared to the methacrylates^55^ was expected to participate more in thiol–ene reactions as opposed to homopolymerization (ene–ene reactions). Moreover, homopolymerization of the grafted methacrylated silane modifier, hyMPS, was hampered by its reduced mobility. This allowed the hyMPS to participate in thiol–ene reactions in a comparable manner with the grafted thiol modifier, hyMPTMS, specifically when their similar molecular structures are considered. Therefore, the more dominant, observable effect on photopolymerization kinetics in this material system was attributed to the concentration of TOCNFs and is initially described, which is then followed by the types of functional surface modifiers. In addition, the effects of methacrylate and thiol modifiers on photopolymerization kinetics could be monitored in isolation, despite the complexity of the reaction system. Finally, the previously mentioned slow solvent evaporation rate of the higher TOCNFs-loaded resins (5 and 10 vol %) restricted the measurement of photocuring kinetics regarding these resins. Without inhibitors, the fast and oxygen-tolerant nature of the thiol–ene chemistry would start uncontrolled polymerization reactions during the removal of solvent, whereas adding inhibitors would directly interfere with the photopolymerization kinetics.

The photo-DSC thermograms of the reference resin and the two composite resins with 1 vol % loading of hyMPS and hyMPTMS modified TOCNFs are displayed in Figures 3c and S19. For all formulations, photo-cross-linking took place within a few minutes under the low irradiance of 0.5 mW/cm^2^, with a peak reaction rate after about 20 s. When comparing the durations required to reach approximately the end of the reaction based on a chosen 0.1 W/g heat flow limit, it was evident that the formulation with 1 vol % Bu_4_NOH-hyMPTMS-TOCNFs needed a longer time (231 s) than other formulations (129 s), as reported in Table 1. Notice that, based on the energy dose equivalence, the light dose of 231 s at 0.5 mW/cm^2^ in photo-DSC corresponded to roughly 8 s under 15 mW/cm^2^, as in the photorheometry measurement, so that the present results were consistent with the data shown in Figure 3b. The geltime associated with the heat flow peak, beyond which the reaction becomes diffusion-controlled, was also slightly shorter for the composite resins. This result was again consistent with the evidence for the gelled nature of the initial suspensions revealed in Figure 3b. The overall reduced conversion of the hyMPTMS-TOCNFs resin might have resulted from light scattering effects of condensed hyMPTMS entities,^45^ as suggested by the Si–O–Si peak formation at 1035 cm^–1^ and relatively weaker −OH band at 3400 cm^–1^ in the FTIR spectrum (Figure 2a). These entities would moreover show decreased mobility and increased steric hindrance, rendering grafting more difficult, as well as hampering the resin access for the surrounding unreacted functionalities on TOCNFs.^56^

**Table 1:** Values Obtained from Photo-DSC Measurements of Reference and Nanocomposite Resins

composite resin type	peak value (W/g)	time to peak (s)	total reaction time (s)^a^	peak area (J/g)
reference (without filler)	7.7	20.4	129	267.3
1 vol % Bu_4_NOH-hyMPS-TOCNFs	7.3	17.4	129	265.2
1 vol % Bu_4_NOH-hyMPTMS-TOCNFs	4.0	18.7	231	219.1

a Time to reach the chosen 0.1 W/g heat flow.

### 3.4 Thermal Stability

The thermogravimetric analysis (Figure 3d) showed that dried as received TOCNFs were characterized with an onset temperature (*T*_onset_, extrapolated onset determined with the beginning of weight loss as the baseline and the tangent line of the inflection point) of 212 °C for thermal degradation following dehydration. The TEMPO-mediated oxidation of cellulose significantly reduced the thermal stability of cellulose due to the small size of the fibrils with a larger surface-to-volume ratio and the decarboxylation reaction during degradation.^57^ In fact, the overall degradation profile of the as received TOCNFs was well-correlated with the previous reports on nanocellulose degradation.^58,59^ Moreover, the introduction of tetrabutylammonium onto the TOCNFs surface drastically reduced the overall thermal stability as well, shifting the 212 °C onset to 179 °C. This performance loss could be associated with the butyl groups connected to the central nitrogen atom by weak C–N bonds. Such a change could be understood based on the fact that the decarboxylation temperature as well as the percentage of char was subject to severe changes based on the countercation species of the carboxylate.^58,59^ The cured reference resin demonstrated a higher thermal stability (*T*_onset_ = 355 °C) than the TOCNFs and underwent a two-step degradation, as also reported with other thiol–enes,^60^ compared to the five steps observed with TOCNFs (Figure S20). The thermal stability of the UV-cured nanocomposite film (1 vol % Bu_4_NOH-hyMPS-TOCNFs) was only mildly affected by the presence of TOCNFs, maintaining a *T*_onset_ comparable to the reference polymer (343 °C). Moreover, the nanocomposite possessed a more pronounced DTG shoulder peak at 455 °C as opposed to the 465 °C shoulder peak observed with the reference thiol–ene polymer, whose increase could be related to siloxane pyrolysis.^61^ Overall, the nanocomposite had a lower degradation rate than the reference above ∼360 °C. The higher thermal stability of siloxanes, the nanofibril structure, as well as the grafted methacrylate groups creating a covalently bonded particle–matrix interface were acknowledged for the improved thermal resistance. The nanofibrils and their surface modifications effectively limited the thermal mobility of the polymer, enhanced the interactions at the particle–matrix interface, and raised the degradation activation energy (bond dissociation).^62,63^

### 3.5 Mechanical Performance

**Figure 4:**
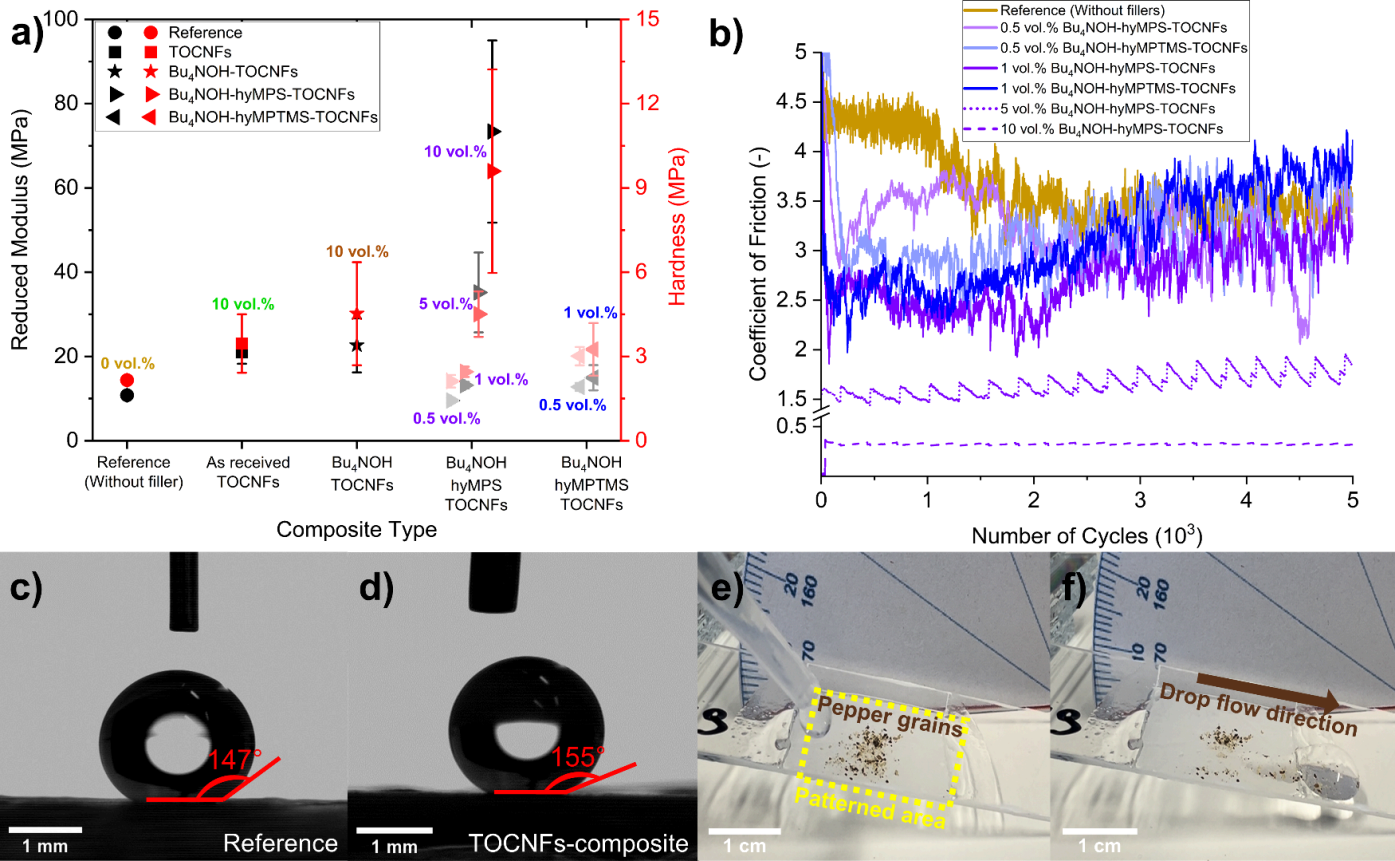
(a) Mechanical performance of cured films in terms of reduced modulus and surface hardness. (b) Evolution of the coefficient of friction in composites at a normal force of 10 mN and 1 Hz frequency. (c and d) Images of 8 μL water drops on the surface of lotus leaf nanopatterned films with cured reference resin (average WCA 139°) and composite resin −0.5 vol % Bu_4_NOH-hyMPS-TOCNFs (average WCA > 150°). (e) Lotus leaf nanopatterned composite film (0.5 vol % Bu_4_NOH-hyMPTMS-TOCNFs as filler) contaminated by pepper grains on the surface. (f) The sliding water drop capturing pepper grains and leaving a clean trace behind.

As shown in Figure 4a and Table 2, which regroup the results from the nanoindentation experiments, the inclusion of TOCNFs inside the thiol–ene resin showed constant improvement in the material surface hardness and reduced modulus. It was even possible to achieve an ∼6 times more reduced modulus and ∼3 times higher surface hardness than the reference resin with the addition of 10 vol % of modified TOCNFs. Such remarkable increases were also reported by Zhu and Dufresne involving natural rubber.^26^ The rise in the performance of the material up to at least 10 vol % of TOCNFs fillers did not reach a peak and potentially has room to further increase with the fiber aspect ratio being a candidate for fine-tuning mechanical properties through percolation.^64^ This benefit will eventually be limited by an agglomeration-free maximum solid loading^65^ or even by porosity concerning pastes^66^ due to insufficient fiber wetting.^67^ The indentation results also clearly demonstrated the considerable effect of surface modification of the TOCNFs. Most notably, with only 1 vol % of Bu_4_NOH-hyMPTMS-TOCNFs the network was as hard as in the case of the composite with 10 vol % unmodified TOCNFs. Such a comparison confirmed the benefits of better nanofiller dispersion and a denser, cross-linked filler–matrix interface for the material performance.^67^ This was reminiscent of the thiol-grafted cellulose nanocrystals in a thiol–ene matrix.^18^ Interestingly, the hyMPTMS-modified TOCNFs showed better performance compared to the hyMPS-modified TOCNFs at least for the particle loadings of 0.5 and 1 vol %. The reason for this was ascribed to the slow curing of resins with thiol-modified TOCNFs as evidenced by the photorheometry and photo-DSC data, because the lower curing speed might have resulted in a more densely cross-linked network^68^ overcoming the mechanical disadvantage of having more thiols compared to the hyMPS variety.

**Table 2:** Reduced Modulus and Surface Hardness of the Cured Reference Resin and Nanocomposite Films with Relative Percentage Variations in Properties in Comparison with the Reference Resin

composite filler type	CNF conc. (vol %)	reduced modulus (MPa)	change (%)	surface hardness (MPa)	change (%)
reference (without filler)	0	10.8 ± 0.3	-	2.2 ± 0.1	-
Bu_4_NOH-hyMPS-TOCNFs	0.5	9.6 ± 0.6	–11	2.1 ± 0.2	–5
Bu_4_NOH-hyMPTMS-TOCNFs	0.5	12.8 ± 0.9	19	3.0 ± 0.3	36
Bu_4_NOH-hyMPS-TOCNFs	1	13.2 ± 0.8	22	2.5 ± 0.2	14
Bu_4_NOH-hyMPTMS-TOCNFs	1	15.0 ± 3.0	39	3.3 ± 0.9	50
Bu_4_NOH-HYMPS-TOCNFs	5	35.2 ± 9.5	226	4.5 ± 0.2	105
As received TOCNFs	10	21.0 ± 2.7	94	3.5 ± 1.0	59
Bu_4_NOH-TOCNFs	10	22.6 ± 6.4	109	4.5 ± 1.8	105
Bu_4_NOH-hyMPS-TOCNFs	10	73.4 ± 21.6	580	9.6 ± 3.6	336

Figure 4b shows the evolving coefficient of friction (CoF) of the composite coatings obtained during oscillatory wearing against a steel ball for 5000 cycles at a 10 mN normal force and 1 Hz. The average CoF values (total of three identical tests per coating) and further wear analysis details of the reference as well as 0.5, 1, 5, and 10 vol % loaded composites are reported in Table 3. The reference coating displayed a two-step CoF decrease with the second decrease occurring from CoF > 4 after ∼1000 cycles, which was reproducible (Figure S21). Adding nanocellulose systematically lowered the CoF. For concentrations of 0.5% and 1%, an initial drop was evident, due to the rubbery nature of the thiol–enes with a low yield strength.^69^ The initial decrease was followed by an increasing trend, with a CoF around 3.5, due to the scraping of a trench on the surface as the friction mechanism. The significantly reduced CoF was ascribed both to the reinforcement of nanofibers and the observed higher roughness of the surface.^69,70^ Moreover, 1 vol % loaded coatings had in the first 2000 cycles lower CoFs compared to the reference. However, the values became similar after 5000 cycles. The CoF also mostly decreased in case of higher normal load (20 mN) and higher frequency (5 Hz), as shown in Figure S22. The effect of higher frequency could be explained with the reduced and inconsistent interactions as well as the possible separations of the counterpart surface with the coating surface.^71^

**Table 3:** Wear Analysis of the Reference and Composite (Bu_4_NOH-hyMPS-TOCNFs) Coatings after 5000 Cycles

composite type	normal force (mN)	coefficient of friction (−)	frictional stress (MPa)	wear volume (10^–3^ mm^3^)	specific wear rate (10^–3^ mm^3^/Nm)
reference (without filler)	10	3.90 ± 0.21	1.00 ± 0.05	0.20 ± 0.03	1.00 ± 0.15
reference (without filler)^a^	10	3.05 ± 0.32	0.78 ± 0.25	0.49 ± 0.30	2.45 ± 1.5
0.5 vol % Bu_4_NOH-hyMPS-TOCNFs	10	3.42 ± 0.14	0.81 ± 0.03	0.33 ± 0.11	1.65 ± 0.55
1 vol % Bu_4_NOH-hyMPS-TOCNFs	10	3.16 ± 0.31	0.92 ± 0.09	2.33 ± 0.63	11.70 ± 3.19
5 vol % Bu_4_NOH-hyMPS-TOCNFs	10	1.05 ± 0.53	0.59 ± 0.30	below detection limit	below detection limit
10 vol % Bu_4_NOH-hyMPS-TOCNFs	10	0.36 ± 0.03	0.33 ± 0.03	below detection limit	below detection limit
reference (without filler)	20	2.78 ± 0.02	0.90 ± 0.01	2.37 ± 1.59	5.93 ± 3.98
0.5 vol % Bu_4_NOH-hyMPS-TOCNFs	20	2.65 ± 0.16	0.79 ± 0.05	9.88 ± 1.32	24.7 ± 3.30
1 vol % Bu_4_NOH-hyMPS-TOCNFs	20	2.53 ± 0.1	0.93 ± 0.04	10.74 ± 3.65	26.86 ± 9.12
5 vol % Bu_4_NOH-hyMPS-TOCNFs	20	0.71 ± 0.39	0.50 ± 0.28	below detection limit	below detection limit
10 vol % Bu_4_NOH-hyMPS-TOCNFs	20	0.30 ± 0.06	0.35 ± 0.07	below detection limit	below detection limit
10 vol % Bu_4_NOH-hyMPS-TOCNFs^a^	300	0.30	0.86	1.37 × 10^–2^	2.28 × 10^–3^
10 vol % Bu_4_NOH-hyMPS-TOCNFs^a^	500	0.25	0.83	5.42 × 10^–2^	5.42 × 10^–3^

a At 5 Hz.

The 5 vol % loaded coating showed a much lower CoF around 1.5, with a response resembling the atomic-level stick–slip behavior observable with atomic force microscopy.^72^ This was likely due to the worn debris altering the wear track that were ultimately pushed out after a threshold force was reached after hundreds of cycles. Remarkably, the CoF of the 10 vol % loaded composites was as low as ∼0.3, indicating more than a 10-fold decrease compared with the unfilled resin, and it remained stable throughout the test, demonstrating the improved robustness of the material. For all coatings, the CoF decreased with increasing normal force, and a similar ∼90% decrease was achieved for the 10 vol % loaded composite compared with the neat polymer under 20 mN of normal force.

Wear loss data are reported in Table 3 in terms of wear volume. The addition of a low (0.5 vol %) concentration of nanocellulose exacerbated the total wear loss, in agreement with a previous study involving micro/nanoparticles inside thermoplastic polyurethane,^73^ especially at a higher normal force. At higher concentrations (>5 vol %), the coatings became much more robust and did not show significant wear (Figure S23). The most impressive results were obtained with the 10 vol % Bu_4_NOH-hyMPS-TOCNFs loaded coating, which showed close-to-none wear loss even at high normal forces such as 300 and 500 mN (Figure S23). No correlation between surface chemistry (MPS/MPTMS) and wear loss was observed. The frictional stresses and specific wear rates provided in Table 3 were calculated as described in ref (73). The decrease in the frictional stresses with increasing nanocellulose was noteworthy and was accompanied by the decreasing contact area owing to the rising reduced modulus. There was no measurable wear depth starting from 5 vol % nanocellulose concentrations for the 10 and 20 mN loads. So a frictional stress ∼0.6 MPa could act as a safety limit below which the contact lifetime could be longer due to avoiding low cycle fatigue and staying within the pure elastic contact regime.^73^

### 3.6 Hydrophobicity of Flat and UVNIL Patterned Composite Coatings

The WCA values of the flat and UVNIL patterned surfaces are listed in Table 4. Notice that it was not possible to produce imprinted nanocomposite films with a TOCNFs concentration greater than 0.5 vol % without defects due to the challenging peel off process. This imperfect peel off was identified under SEM by the broken residual composite film parts on the PDMS surface (Figure S25) and was attributed to the reduced ductility of the composite and local stress concentrations. The reference resin was slightly hydrophobic (78° WCA), whereas the introduction of 0.5 vol % of modified cellulose nanofibrils increased hydrophobicity (about 85–90° WCA) owing to both the increase in surface roughness (Figures S6 and S24) and reduced polarity due to Bu_4_NOH and hyMPS/hyMPTMS modifications. The replication of the nanostructured surface of the lotus leaf resulted in WCA values above 150° (Figures 4c and 4d), a laudable value given the hydrophilic nature of cellulose and the absence of any surfactant, in particular fluorine-based. During WCA measurements, it was challenging to place the few microliters of water droplets, which were sliding on the patterned surfaces (Video S1). In addition, the self-cleaning capability of the coatings was proven by means of applying water drops to the surface contaminated with hydrophobic pepper grains. Specifically, the water drops started to slide when the patterned composite films with 0.5 vol % Bu_4_NOH-hyMPS-TOCNFs and 0.5 vol % Bu_4_NOH-hyMPTMS-TOCNFs were tilted by 25° and 9° respectively, and captured the hydrophobic pepper contaminants (Figures 4e and 4f and Video S2). The latter film having a WCA > 150° and a sliding angle <10° meets the criteria of being a superhydrophobic surface.^12,74^ Overall, considering that the coatings were not functionalized with fluorine, which is the standard for increasing hydrophobicity and self-cleaning properties,^36^ the presented results look promising toward a potential deviation from fluorinated polymers for hydrophobicity.

**Table 4:** Water Contact Angle Results Calculated with Five Measurements Per Resin and Surface Type

	WCA	
resin type	flat surface (deg)	lotus patterned (deg)
reference (without filler)	78.1 ± 0.8	139 ± 8
0.5 vol % Bu_4_NOH-hyMPS-TOCNFs	87.6 ± 0.3	150.3 ± 3
0.5 vol % Bu_4_NOH-hyMPTMS-TOCNFs	86.6 ± 0.7	150.6 ± 1
1 vol % Bu_4_NOH-hyMPS-TOCNFs	83.8 ± 0.8	-
1 vol % Bu_4_NOH-hyMPTMS-TOCNFs	89.5 ± 0.6	-

**Figure 5:**
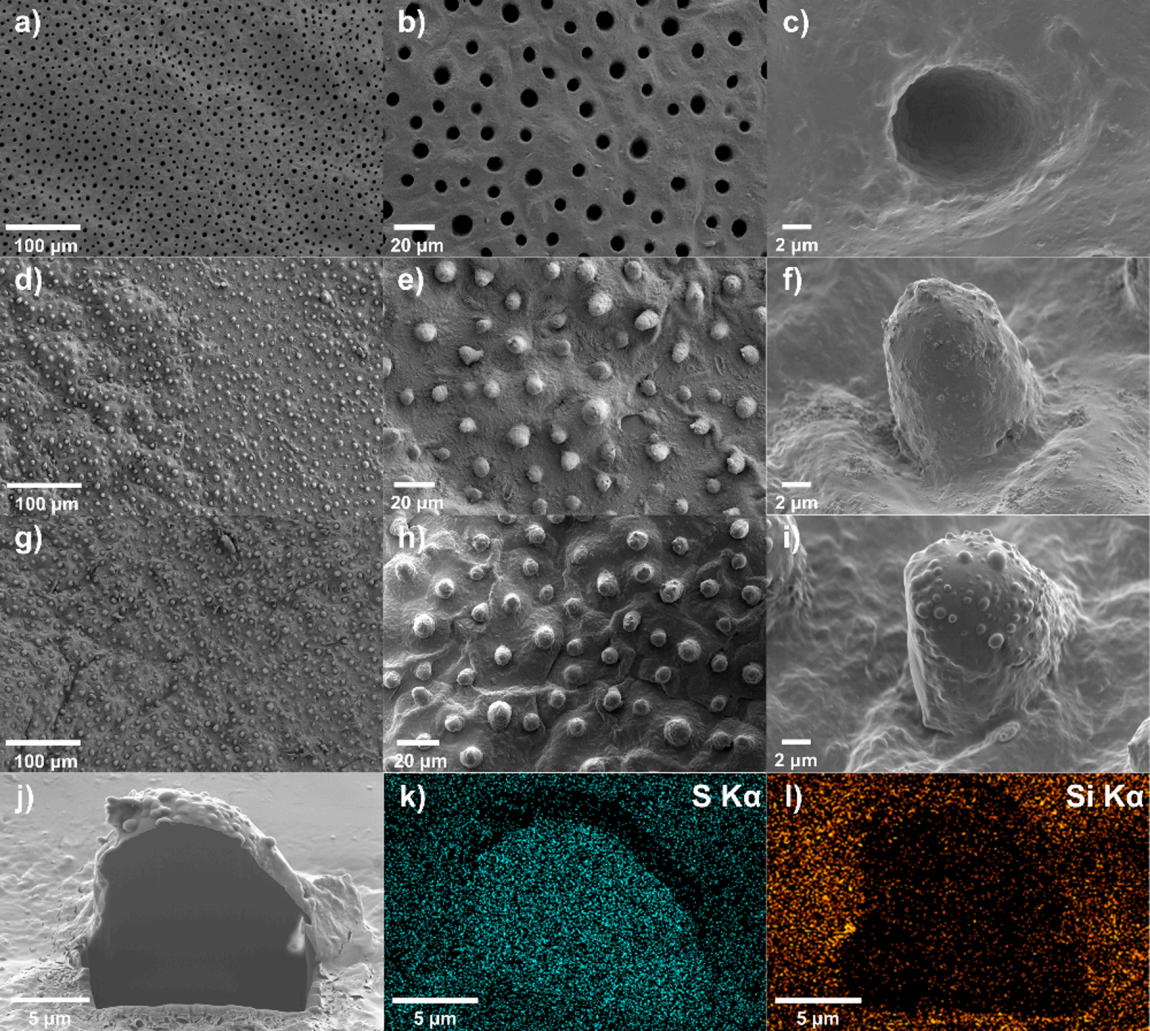
Electron microscopy images of the PDMS negative replica of the lotus leaf hierarchical surface (a–c), UVNIL imprinted reference resin film (d–f), UVNIL imprinted 0.5 vol % Bu_4_NOH-hyMPS-TOCNFs nanocomposite resin film (g–i) at three magnification levels (150×, 600×, and 4000×), and milled cross section of one of the replicated micropapillae (j) and corresponding elemental mapping of S (thiol–ene network, k) and Si (Bu_4_NOH-hyMPS-TOCNFs, l).

Scanning electron micrographs of the texturized surfaces (Figure 5) revealed that the PDMS mold was able to negatively replicate the micro/nanostructured surface of the lotus leaf.^3^ However, the positive replication of the reference resin film was not completely successful, since the nanometric features on the surface of the micropapillae were not well reproduced (Figure 5f) and the surface morphology contains numerous flat regions. On the contrary, the nanoimprinted nanocomposite surface with 0.5 vol % Bu_4_NOH-hyMPS-TOCNFs exhibited submicron bumps at the surface of the micropapillae (Figure 5i). The bumps-on-bumps morphology of the composite film did replicate the double-roughness of actual lotus micropapillae (1.7–19 μm) and their needle-shaped wax tubes on top (0.3–1.7 μm).^75^ This morphology was investigated by milling one of the bumps via a Ga^+^ focused ion beam (Figures 5j–5l). Elemental mapping of S (Figure 5k) and Si (Figure 5l) revealed the homogeneity of the polymer network and the evenly distributed TOCNFs inside the polymer matrix, respectively. The weaker Si signal within the replicated papillae would correspond to a reduced concentration of TOCNFs, which may have resulted from a flow-induced resin enrichment during the imprinting process. Nevertheless, the submicron bumps were the replica of the lotus wax tubes and not clusters formed by preferentially accumulating TOCNFs at the PDMS walls.

## 4 Conclusions

Photocurable nanocellulose/thiol–ene composite resins were formulated to produce composite coatings. Attention was paid to the role of the cellulose surface treatment on the rheological behavior of nanocellulose suspensions, curing kinetics and mechanical performance of the cured composites. The composite resins were finally utilized to replicate lotus leaf surfaces to create self-cleaning surfaces. The three most significant processing challenges, namely the solvent evaporation after mixing the thiol–ene phase with nanocellulose, the high viscosity of the nanocellulose suspensions, and the dispersion of the nanocellulose particles, were investigated and limitations as well as methods of improvement were described. The solvent evaporation, which was found to be practically limited mostly by the time required, was demanding for the TOCNFs loading of 1 vol %. Besides, the seemingly high viscosity can be circumvented during processing of these resins by applying shear (>1 s^–1^) owing to the shear-thinning behavior with power law exponents ∼0.3. And last, the dispersion of TOCNFs inside the thiol–ene resin was achieved through experimentation with a set of surface modifications comprising quaternary alkylammonium counterions for cation exchange at the carboxylate of the TOCNFs and grafting methacrylate and thiol functional groups onto the fibril surface. The number of precursors was kept to a minimum for simplicity, and no fluorine-based surfactants or dispersants were used. The resulting improved dispersion led to transparent films with enhanced mechanical properties. Moreover, the TOCNFs did not impede the initial photopolymerization stage, and the photopolymerization was also completed in almost identical times compared to the reference resin when methacrylate-grafted TOCNFs were used. The added nanocellulose fillers granted up to a 580% increase in reduced modulus and a 336% increase in surface hardness, and for the wear test with parameters of 10 mN loading and 1 Hz frequency, more than 90% decrease of the CoF and full prevention of wear loss were achieved in comparison with the thiol–ene network without fillers. Using a UVNIL process to replicate a lotus leaf, the composite film with 0.5 vol % Bu_4_NOH-hyMPS-modified TOCNFs moreover featured a double-roughness with submicron bumps compared with the neat thiol–ene polymer, leading to a superhydrophobic and self-cleaning behavior while eluding fluorinated polymers. The prepared viscous yet free-flowing nanocellulose/thiol–ene composite resins can be potentially utilized in photopolymerization-assisted production processes with rapid curing like 3D printing and roll-to-roll processing. The exceptional wear resistance of the TOCNF-reinforced thiol–ene composites makes these materials very well suited in applications where elastomeric materials are necessary yet mechanically underperforming, such as soft robotics or packaging of wearable microelectromechanical systems.

## Data Availability

The experimental data are available from the corresponding author upon reasonable request.

## Supporting Information Available

Chemical structures of materials, characterization of resins and cured materials, prehydrolysis of surface modifiers, variation in light transmittance based on filler concentration, improvement in particle dispersion following cation exchange, morphologies and sizes of modified TOCNFs, photopolymerization of thiol–ene reference resin, photocuring of composite resins with changing viscosity and heat flow, thermal degradation of as received TOCNF, tribological analysis of the composite coatings, and contaminated UVNIL pattern template surface following polymer film peel off (PDF)
Video S1, placement of the few microliters of water droplets (MP4)
Video S2, water droplets capturing the hydrophobic pepper contaminants (MOV)

## Supplementary Material

am4c16445_si_002.mp4

am4c16445_si_003.mov

## References

[ref1] Li X.-M.; Reinhoudt D.; Crego-Calama M. What Do We Need for a Superhydrophobic Surface? A Review on the Recent Progress in the Preparation of Superhydrophobic Surfaces. Chem. Soc. Rev. 2007, 36 (8), 1350–1368. 10.1039/b602486f. 17619692

[ref2] Barthlott W.; Neinhuis C. Purity of the Sacred Lotus, or Escape from Contamination in Biological Surfaces. Planta 1997, 202 (1), 1–8. 10.1007/s004250050096.

[ref3] Patankar N. A. Mimicking the Lotus Effect: Influence of Double Roughness Structures and Slender Pillars. Langmuir 2004, 20 (19), 8209–8213. 10.1021/la048629t. 15350093

[ref4] Hoshian S.; Jokinen V.; Somerkivi V.; Lokanathan A. R.; Franssila S. Robust Superhydrophobic Silicon without a Low Surface-Energy Hydrophobic Coating. ACS Appl. Mater. Interfaces 2015, 7 (1), 941–949. 10.1021/am507584j. 25522296

[ref5] Pan Z.; Cheng F.; Zhao B. Bio-Inspired Polymeric Structures with Special Wettability and Their Applications: An Overview. Polymers 2017, 9 (12), 725 10.3390/polym9120725. 30966026 PMC6418807

[ref6] Lohmann R.; Cousins I. T.; DeWitt J. C.; Glüge J.; Goldenman G.; Herzke D.; Lindstrom A. B.; Miller M. F.; Ng C. A.; Patton S.; Scheringer M.; Trier X.; Wang Z. Are Fluoropolymers Really of Low Concern for Human and Environmental Health and Separate from Other PFAS?. Environ. Sci. Technol. 2020, 54 (20), 12820–12828. 10.1021/acs.est.0c03244. 33043667 PMC7700770

[ref7] Poothanari M. A.; Schreier A.; Missoum K.; Bras J.; Leterrier Y. Photocured Nanocellulose Composites: Recent Advances. ACS Sustainable Chem. Eng. 2022, 10 (10), 3131–3149. 10.1021/acssuschemeng.1c07631.

[ref8] Hoyle C. E.; Bowman C. N. Thiol–Ene Click Chemistry. Angew. Chem., Int. Ed. 2010, 49 (9), 1540–1573. 10.1002/anie.200903924. 20166107

[ref9] Reddy S. K.; Cramer N. B.; Cross T.; Raj R.; Bowman C. N. Polymer-Derived Ceramic Materials from Thiol-Ene Photopolymerizations. Chem. Mater. 2003, 15 (22), 4257–4261. 10.1021/cm034291x.

[ref10] Natali M.; Begolo S.; Carofiglio T.; Mistura G. Rapid Prototyping of Multilayer Thiolene Microfluidic Chips by Photopolymerization and Transfer Lamination. Lab Chip 2008, 8 (3), 492–494. 10.1039/b716594c. 18305871

[ref11] Khire V. S.; Yi Y.; Clark N. A.; Bowman C. N. Formation and Surface Modification of Nanopatterned Thiol-Ene Substrates Using Step and Flash Imprint Lithography. Adv. Mater. 2008, 20 (17), 3308–3313. 10.1002/adma.200800672.

[ref12] Resetco C.; Hendriks B.; Badi N.; Du Prez F. Thiol–Ene Chemistry for Polymer Coatings and Surface Modification – Building in Sustainability and Performance. Materials Horizons 2017, 4 (6), 1041–1053. 10.1039/C7MH00488E.

[ref13] Islam M. S.; Masoodi R.; Rostami H. The Effect of Nanoparticles Percentage on Mechanical Behavior of Silica-Epoxy Nanocomposites. Journal of Nanoscience 2013, e275037 10.1155/2013/275037.

[ref14] Omanović-Mikličanin E.; Badnjević A.; Kazlagić A.; Hajlovac M. Nanocomposites: A Brief Review. Health Technol. 2020, 10 (1), 51–59. 10.1007/s12553-019-00380-x.

[ref15] Barnes E.; Jefcoat J. A.; Alberts E. M.; McKechnie M. A.; Peel H. R.; Buchanan J. P.; Weiss Jr. C. A.; Klaus K. L.; Mimun L. C.; Warner C. M. Effect of Cellulose Nanofibrils and TEMPO-Mediated Oxidized Cellulose Nanofibrils on the Physical and Mechanical Properties of Poly(Vinylidene Fluoride)/Cellulose Nanofibril Composites. Polymers 2019, 11 (7), 1091 10.3390/polym11071091. 31252644 PMC6680576

[ref16] Prakasam M.; Locs J.; Salma-Ancane K.; Loca D.; Largeteau A.; Berzina-Cimdina L. Biodegradable Materials and Metallic Implants—A Review. Journal of Functional Biomaterials 2017, 8 (4), 44 10.3390/jfb8040044. 28954399 PMC5748551

[ref17] Weiss M.; Haufe J.; Carus M.; Brandão M.; Bringezu S.; Hermann B.; Patel M. K. A Review of the Environmental Impacts of Biobased Materials. Journal of Industrial Ecology 2012, 16 (s1), S169–S181. 10.1111/j.1530-9290.2012.00468.x.

[ref18] Parambath Kanoth B.; Claudino M.; Johansson M.; Berglund L. A.; Zhou Q. Biocomposites from Natural Rubber: Synergistic Effects of Functionalized Cellulose Nanocrystals as Both Reinforcing and Cross-Linking Agents via Free-Radical Thiol–Ene Chemistry. ACS Appl. Mater. Interfaces 2015, 7 (30), 16303–16310. 10.1021/acsami.5b03115. 26151647

[ref19] Fuenmayor C.; da Silva D. A; Klock U.; Lengowski E. C; de Andrade A. S; Bonfatti E. A; Trejo J.; Nunez Y.; Palma O.; de Cademartori P. H. G; de Muniz G. I. B Tribological Behavior of Cellulose Nanostructured Films. Surf. Topogr.: Metrol. Prop. 2022, 10 (3), 034001 10.1088/2051-672X/ac7fab.

[ref20] Xu X.; Liu F.; Jiang L.; Zhu J. Y.; Haagenson D.; Wiesenborn D. P. Cellulose Nanocrystals vs. Cellulose Nanofibrils: A Comparative Study on Their Microstructures and Effects as Polymer Reinforcing Agents. ACS Appl. Mater. Interfaces 2013, 5 (8), 2999–3009. 10.1021/am302624t. 23521616

[ref21] Bäckström M.; Bolivar S.; Paltakari J. Effect of Ionic Form of Fibrillation and the Development of the Fibre Network Strength during the Refining of the Kraft Pulps. ÓPapel 2012, 73 (7), 57–65.

[ref22] Liu S.; Low Z.-X.; Xie Z.; Wang H. TEMPO-Oxidized Cellulose Nanofibers: A Renewable Nanomaterial for Environmental and Energy Applications. Advanced Materials Technologies 2021, 6 (7), 2001180 10.1002/admt.202001180.

[ref23] Isogai A.; Saito T.; Fukuzumi H. TEMPO-Oxidized Cellulose Nanofibers. Nanoscale 2011, 3 (1), 71–85. 10.1039/C0NR00583E. 20957280

[ref24] Nechyporchuk O.; Belgacem M. N.; Bras J. Production of Cellulose Nanofibrils: A Review of Recent Advances. Industrial Crops and Products 2016, 93, 2–25. 10.1016/j.indcrop.2016.02.016.

[ref25] Kargarzadeh H.; Mariano M.; Huang J.; Lin N.; Ahmad I.; Dufresne A.; Thomas S. Recent Developments on Nanocellulose Reinforced Polymer Nanocomposites: A Review. Polymer 2017, 132, 368–393. 10.1016/j.polymer.2017.09.043.

[ref26] Zhu G.; Dufresne A. Synergistic Reinforcing and Cross-Linking Effect of Thiol-Ene-Modified Cellulose Nanofibrils on Natural Rubber. Carbohydr. Polym. 2022, 278, 118954 10.1016/j.carbpol.2021.118954. 34973770

[ref27] Shimizu M.; Saito T.; Isogai A. Bulky Quaternary Alkylammonium Counterions Enhance the Nanodispersibility of 2,2,6,6-Tetramethylpiperidine-1-Oxyl-Oxidized Cellulose in Diverse Solvents. Biomacromolecules 2014, 15 (5), 1904–1909. 10.1021/bm500384d. 24750066

[ref28] Daicho K.; Kobayashi K.; Fujisawa S.; Saito T. Crystallinity-Independent yet Modification-Dependent True Density of Nanocellulose. Biomacromolecules 2020, 21 (2), 939–945. 10.1021/acs.biomac.9b01584. 31820948

[ref29] Iqbal D.; Zhao Y.; Zhao R.; Russell S. J.; Ning X. A Review on Nanocellulose and Superhydrophobic Features for Advanced Water Treatment. Polymers 2022, 14 (12), 2343 10.3390/polym14122343. 35745924 PMC9229312

[ref30] Hu D.; Ma W.; Zhang Z.; Ding Y.; Wu L. Dual Bio-Inspired Design of Highly Thermally Conductive and Superhydrophobic Nanocellulose Composite Films. ACS Appl. Mater. Interfaces 2020, 12 (9), 11115–11125. 10.1021/acsami.0c01425. 32049475

[ref31] Lin H.; Wan X.; Jiang X.; Wang Q.; Yin J. A Nanoimprint Lithography Hybrid Photoresist Based on the Thiol–Ene System. Adv. Funct. Mater. 2011, 21 (15), 2960–2967. 10.1002/adfm.201100692.

[ref32] Leitgeb M.; Nees D.; Ruttloff S.; Palfinger U.; Götz J.; Liska R.; Belegratis M. R.; Stadlober B. Multilength Scale Patterning of Functional Layers by Roll-to-Roll Ultraviolet-Light-Assisted Nanoimprint Lithography. ACS Nano 2016, 10 (5), 4926–4941. 10.1021/acsnano.5b07411. 27023664

[ref33] Handrea-Dragan I. M.; Botiz I.; Tatar A.-S.; Boca S. Patterning at the Micro/Nano-Scale: Polymeric Scaffolds for Medical Diagnostic and Cell-Surface Interaction Applications. Colloids Surf., B 2022, 218, 112730 10.1016/j.colsurfb.2022.112730. 35932559

[ref34] Lan H. Soft UV Nanoimprint Lithography and Its Applications. In Updates in Advanced Lithography; IntechOpen, 2013. 10.5772/56186.

[ref35] Choo S.; Choi H.-J.; Lee H. Replication of Rose-Petal Surface Structure Using UV-Nanoimprint Lithography. Mater. Lett. 2014, 121, 170–173. 10.1016/j.matlet.2014.01.037.

[ref36] González Lazo M. A.; Katrantzis I.; Dalle Vacche S.; Karasu F.; Leterrier Y. A Facile in Situ and UV Printing Process for Bioinspired Self-Cleaning Surfaces. Materials 2016, 9 (9), 738 10.3390/ma9090738. 28773860 PMC5457055

[ref37] Cully P.; Karasu F.; Müller L.; Jauzein T.; Leterrier Y. Self-Cleaning and Wear-Resistant Polymer Nanocomposite Surfaces. Surf. Coat. Technol. 2018, 348, 111–120. 10.1016/j.surfcoat.2018.05.040.

[ref38] Nagarjuna R.; Saifullah M. S. M.; Ganesan R. Oxygen Insensitive Thiol–Ene Photo-Click Chemistry for Direct Imprint Lithography of Oxides. RSC Adv. 2018, 8 (21), 11403–11411. 10.1039/C8RA01688G. 35542774 PMC9079138

[ref39] Wolfberger A.; Rupp B.; Kern W.; Griesser T.; Slugovc C. Ring Opening Metathesis Polymerization Derived Polymers as Photoresists: Making Use of Thiol-Ene Chemistry. Macromol. Rapid Commun. 2011, 32 (6), 518–522. 10.1002/marc.201000698. 21433209

[ref40] Lu J.; Askeland P.; Drzal L. T. Surface Modification of Microfibrillated Cellulose for Epoxy Composite Applications. Polymer 2008, 49 (5), 1285–1296. 10.1016/j.polymer.2008.01.028.

[ref41] Galland S.; Leterrier Y.; Nardi T.; Plummer C. J. G.; Månson J. A. E.; Berglund L. A. UV-Cured Cellulose Nanofiber Composites with Moisture Durable Oxygen Barrier Properties. J. Appl. Polym. Sci. 2014, 131 (16), n/a 10.1002/app.40604.

[ref42] Esfandiari P.; Ligon S. C.; Lagref J. J.; Frantz R.; Cherkaoui Z.; Liska R. Efficient Stabilization of Thiol-Ene Formulations in Radical Photopolymerization. J. Polym. Sci., Part A: Polym. Chem. 2013, 51 (20), 4261–4266. 10.1002/pola.26848.

[ref43] Neves A. D.; Discacciati J. a. C.; Oréfice R. L.; Yoshida M. I. Influence of the Power Density on the Kinetics of Photopolymerization and Properties of Dental Composites. Journal of Biomedical Materials Research Part B: Applied Biomaterials 2005, 72B (2), 393–400. 10.1002/jbm.b.30179. 15654701

[ref44] Jiang F.; Han S.; Hsieh Y.-L. Controlled Defibrillation of Rice Straw Cellulose and Self-Assembly of Cellulose Nanofibrils into Highly Crystalline Fibrous Materials. RSC Adv. 2013, 3 (30), 12366–12375. 10.1039/c3ra41646a.

[ref45] Rahman I. A.; Padavettan V. Synthesis of Silica Nanoparticles by Sol-Gel: Size-Dependent Properties, Surface Modification, and Applications in Silica-Polymer Nanocomposites—A Review. J. Nanomater. 2012, 2012, e132424 10.1155/2012/132424.

[ref46] Rachini A.; Le Troedec M.; Peyratout C.; Smith A. Chemical Modification of Hemp Fibers by Silane Coupling Agents. J. Appl. Polym. Sci. 2012, 123 (1), 601–610. 10.1002/app.34530.

[ref47] Johan Foster E.; Moon R. J.; Agarwal U. P.; Bortner M. J.; Bras J.; Camarero-Espinosa S.; Chan J. K.; Clift M. J. D.; Cranston E. D.; Eichhorn S. J.; Fox D. M.; Hamad W. Y.; Heux L.; Jean B.; Korey M.; Nieh W.; Ong K. J.; Reid M. S.; Renneckar S.; Roberts R.; Anne Shatkin J.; Simonsen J.; Stinson-Bagby K.; Wanasekara N.; Youngblood J. Current Characterization Methods for Cellulose Nanomaterials. Chem. Soc. Rev. 2018, 47 (8), 2609–2679. 10.1039/C6CS00895J. 29658545

[ref48] Väisänen S.; Pönni R.; Hämäläinen A.; Vuorinen T. Quantification of Accessible Hydroxyl Groups in Cellulosic Pulps by Dynamic Vapor Sorption with Deuterium Exchange. Cellulose 2018, 25 (12), 6923–6934. 10.1007/s10570-018-2064-0.

[ref49] Jonasson S.; Bünder A.; Niittylä T.; Oksman K. Isolation and Characterization of Cellulose Nanofibers from Aspen Wood Using Derivatizing and Non-Derivatizing Pretreatments. Cellulose 2020, 27 (1), 185–203. 10.1007/s10570-019-02754-w.

[ref50] Lundahl M. J.; Cunha A. G.; Rojo E.; Papageorgiou A. C.; Rautkari L.; Arboleda J. C.; Rojas O. J. Strength and Water Interactions of Cellulose I Filaments Wet-Spun from Cellulose Nanofibril Hydrogels. Sci. Rep 2016, 6 (1), 30695 10.1038/srep30695. 27465828 PMC4964603

[ref51] Jakubek Z. J.; Chen M.; Couillard M.; Leng T.; Liu L.; Zou S.; Baxa U.; Clogston J. D.; Hamad W. Y.; Johnston L. J. Characterization Challenges for a Cellulose Nanocrystal Reference Material: Dispersion and Particle Size Distributions. J. Nanopart. Res. 2018, 20 (4), 98 10.1007/s11051-018-4194-6.

[ref52] Mendoza L.; Batchelor W.; Tabor R. F.; Garnier G. Gelation Mechanism of Cellulose Nanofibre Gels: A Colloids and Interfacial Perspective. J. Colloid Interface Sci. 2018, 509, 39–46. 10.1016/j.jcis.2017.08.101. 28881204

[ref53] Hoyle C. E.; Lee T. Y.; Roper T. Thiol–Enes: Chemistry of the Past with Promise for the Future. J. Polym. Sci., Part A: Polym. Chem. 2004, 42 (21), 5301–5338. 10.1002/pola.20366.

[ref54] Cole M. A.; Jankousky K. C.; Bowman C. N. Redox Initiation of Bulk Thiol–Ene Polymerizations. Polym. Chem. 2013, 4 (4), 1167–1175. 10.1039/C2PY20843A. 23565125 PMC3616679

[ref55] Northrop B. H.; Coffey R. N. Thiol–Ene Click Chemistry: Computational and Kinetic Analysis of the Influence of Alkene Functionality. J. Am. Chem. Soc. 2012, 134 (33), 13804–13817. 10.1021/ja305441d. 22853003

[ref56] Moretto E.; Fernandes J. P. C.; Staropoli M.; Rogé V.; Steiner P.; Duez B.; Lenoble D.; Thomann J.-S. Dual-Silane Premodified Silica Nanoparticles–Synthesis and Interplay between Chemical, Mechanical, and Curing Properties of Silica–Rubber Nanocomposites: Application to Tire Tread Compounds. ACS Omega 2022, 7 (21), 17692–17702. 10.1021/acsomega.2c00665. 35664568 PMC9161251

[ref57] Mahendra I. P.; Wirjosentono B.; Tamrin I. H.; Mendez J. A. Thermal and Morphology Properties of Cellulose Nanofiber from TEMPO-Oxidized Lower Part of Empty Fruit Bunches (LEFB). Open Chemistry 2019, 17 (1), 526–536. 10.1515/chem-2019-0063.

[ref58] Lichtenstein K.; Lavoine N. Toward a Deeper Understanding of the Thermal Degradation Mechanism of Nanocellulose. Polym. Degrad. Stab. 2017, 146, 53–60. 10.1016/j.polymdegradstab.2017.09.018.

[ref59] Fukuzumi H.; Saito T.; Okita Y.; Isogai A. Thermal Stabilization of TEMPO-Oxidized Cellulose. Polym. Degrad. Stab. 2010, 95 (9), 1502–1508. 10.1016/j.polymdegradstab.2010.06.015.

[ref60] Chen H.; Zhu S.; Zhou R.; Wu X.; Zhang W.; Han X.; Wang J. Thermal Degradation Behavior of Thiol-Ene Composites Loaded with a Novel Silicone Flame Retardant. Polymers 2022, 14 (20), 4335 10.3390/polym14204335. 36297910 PMC9610742

[ref61] Loof D.; Hiller M.; Oschkinat H.; Koschek K. Quantitative and Qualitative Analysis of Surface Modified Cellulose Utilizing TGA-MS. Materials 2016, 9 (6), 415 10.3390/ma9060415. 28773537 PMC5456835

[ref62] Gan P. G.; Sam S. T.; Abdullah M. F. b.; Omar M. F. Thermal Properties of Nanocellulose-Reinforced Composites: A Review. J. Appl. Polym. Sci. 2020, 137 (11), 48544 10.1002/app.48544.

[ref63] Bikiaris D. Can Nanoparticles Really Enhance Thermal Stability of Polymers? Part II: An Overview on Thermal Decomposition of Polycondensation Polymers. Thermochim. Acta 2011, 523 (1), 25–45. 10.1016/j.tca.2011.06.012.

[ref64] Hubbe M. A.; Rojas O. J.; Lucia L. A.; Sain M. Cellulosic Nanocomposites, Review. BioResources 2008, 3 (3), 929–980.

[ref65] Krieger I. M.; Dougherty T. J. A Mechanism for Non-Newtonian Flow in Suspensions of Rigid Spheres. Transactions of the Society of Rheology 1959, 3 (1), 137–152. 10.1122/1.548848.

[ref66] Ez-zaki H.; Riva L.; Bellotto M.; Valentini L.; Garbin E.; Punta C.; Artioli G. Influence of Cellulose Nanofibrils on the Rheology, Microstructure and Strength of Alkali Activated Ground Granulated Blast-Furnace Slag: A Comparison with Ordinary Portland Cement. Mater. Struct 2021, 54 (1), 23 10.1617/s11527-020-01614-5.

[ref67] Abdelmouleh M.; Boufi S.; Belgacem M. N.; Dufresne A.; Gandini A. Modification of Cellulose Fibers with Functionalized Silanes: Effect of the Fiber Treatment on the Mechanical Performances of Cellulose–Thermoset Composites. J. Appl. Polym. Sci. 2005, 98 (3), 974–984. 10.1002/app.22133.

[ref68] Van de Voorde K. The Effect of Cure Rate on Glassy Polymer Networks. Honors College Thesis, The University of Southern Mississippi, Hattiesburg, MS, 2016. https://aquila.usm.edu/honors_theses/361.

[ref69] Wang R.-M.; Zheng S.-R.; Zheng Y.-P. 11 - Other Properties of Polymer Composites. In Polymer Matrix Composites and Technology; Wang R.-M., Zheng S.-R., Zheng Y.-P., Eds.; Woodhead Publishing Series in Composites Science and Engineering; Woodhead Publishing, 2011; pp 513–548. 10.1533/9780857092229.3.513.

[ref70] Manhart J.; Hausberger A.; Mühlbacher I.; Schaller R.; Holzner A.; Kern W.; Schlögl S. UV-Induced Modulation of Tribological Characteristics: Elastomeric Materials Featuring Controlled Anisotropic Friction Properties. AIP Conf. Proc. 2016, 1779 (1), 080004 10.1063/1.4965548.

[ref71] Chowdhury M. A.; Helali Md. M. The Effect of Frequency of Vibration and Humidity on the Coefficient of Friction. Tribiol. Int. 2006, 39 (9), 958–962. 10.1016/j.triboint.2005.10.002.

[ref72] Carpick R. W.; Martini A.; Cannara R. J. Atomic-Level Stick-Slip. In Encyclopedia of Tribology; Wang Q. J., Chung Y.-W., Eds.; Springer US: Boston, MA, 2013; pp 140–148. 10.1007/978-0-387-92897-5_509.

[ref73] Golaz B.; Tetouani S.; Diomidis N.; Michaud V.; Mischler S. Processing and Tribology of Thermoplastic Polyurethane Particulate Composite Materials. J. Appl. Polym. Sci. 2012, 125 (5), 3745–3754. 10.1002/app.36543.

[ref74] Shang Q.; Zhou Y. Fabrication of Transparent Superhydrophobic Porous Silica Coating for Self-Cleaning and Anti-Fogging. Ceram. Int. 2016, 42 (7), 8706–8712. 10.1016/j.ceramint.2016.02.105.

[ref75] Yamamoto M.; Nishikawa N.; Mayama H.; Nonomura Y.; Yokojima S.; Nakamura S.; Uchida K. Theoretical Explanation of the Lotus Effect: Superhydrophobic Property Changes by Removal of Nanostructures from the Surface of a Lotus Leaf. Langmuir 2015, 31 (26), 7355–7363. 10.1021/acs.langmuir.5b00670. 26075949

