# Supplementary material for: Photocurable Thiol–Ene/Nanocellulose Elastomeric Composites for Bioinspired and Fluorine-Free Superhydrophobic Surfaces
Source: ACS Appl Mater Interfaces. 2024 Oct 24;16(44):61144–56. doi: 10.1021/acsami.4c16445 (PMC11551906; doi:10.1021/acsami.4c16445)
Supplement: Supplementary file 1 — am4c16445_si_001.pdf [file am4c16445_si_001.pdf]

Supporting Information

Photo-curable thiol-ene / nanocellulose elastomeric composites for bio-inspired and fluorine-free superhydrophobic surfaces

Alper Balkan ‡, Enrico Sola ‡, Feyza Karasu and Yves Leterrier*

Laboratory for Processing of Advanced Composites (LPAC)

École Polytechnique Fédérale de Lausanne (EPFL)

CH-1015 Lausanne, Switzerland

‡ A.B. and E.S. contributed equally to this paper.

*Corresponding author, yves.leterrier@epfl.ch

Chemical structures of materials

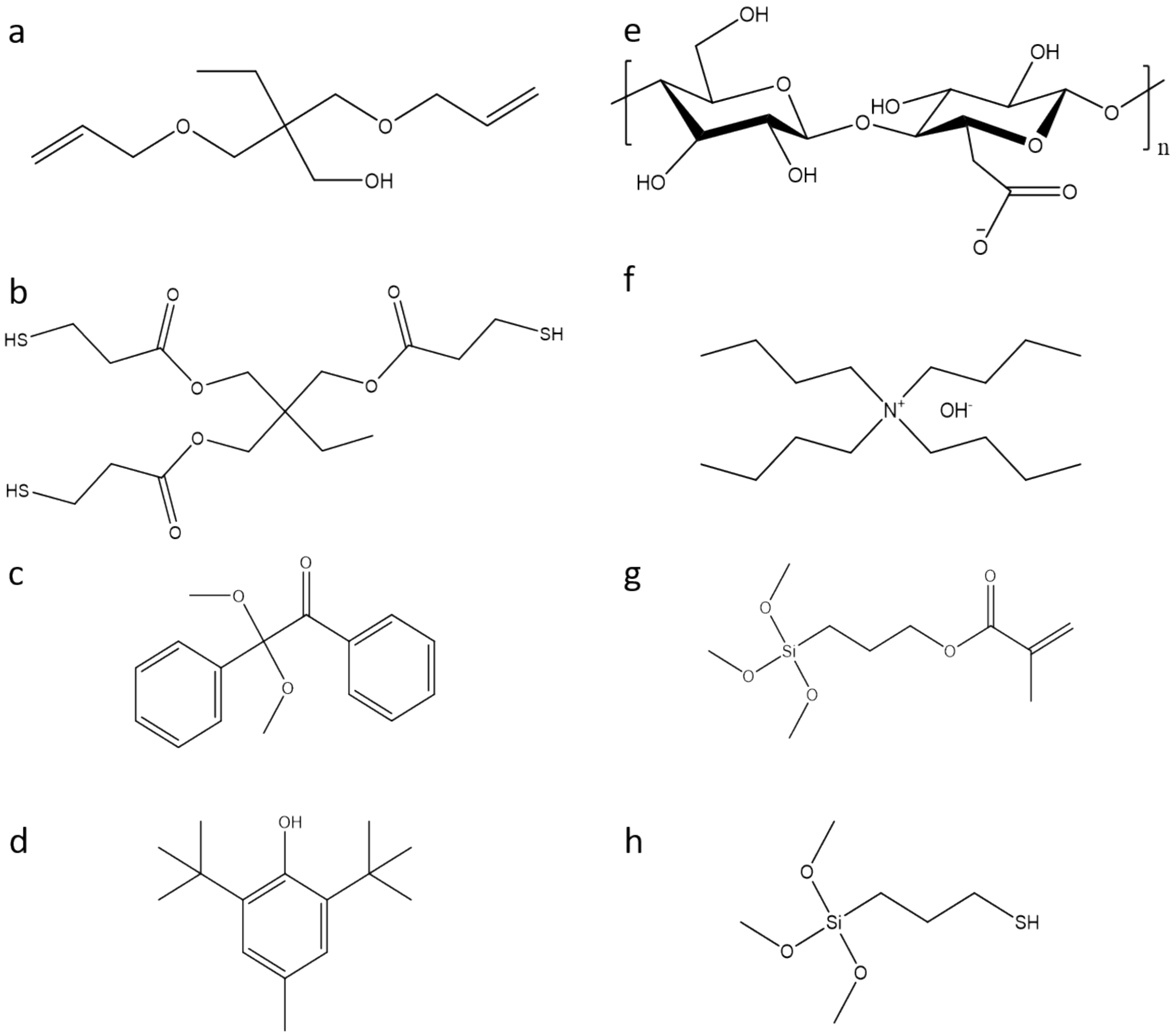

Figure S1. Molecular structures of resin components including (a) TMPDE, (b) TMPTMP, (c) DMPA, (d) BHT, (e) TOCNFs without counter cation, (f) Bu_4NOH, (g) MPS, (h) MPTMS.

Characterization of resins and cured materials

The Nicolet 6700 FT-IR Spectrometer (Thermo Fisher Scientific, USA) was used to evaluate the thiol-ene reaction between TMPDE and TMPTMP, and the effectiveness of TOCNFs surface modifications. Dried cellulose nanofibril samples were prepared by means of KBr disks dried inside an oven at 120 °C for 2 days. Additionally, TOCNFs and Bu_4NOH-functionalized TOCNFs were characterized by zeta potential measurements within water as dispersion medium (particle concentration: 0.1 wt.%) using a 633 nm laser (Zetasizer Nano ZS, Malvern, UK). Atomic force microscopy (Cypher VRS, Oxford Instruments Asylum Research, UK) was done in tapping mode in air by antimony-doped silicon cantilevers with aluminum reflex coating (Spring constant k = 2.8 N/m, nominal tip radius: 8 nm, operated at approx. 75 kHz). The samples were prepared by pipetting a drop of 1-0.5 wt.% suspension onto a freshly cleaved muscovite mica (V-1 Q, Electron Microscopy Sciences, USA) after being dried for a day at room temperature. The obtained AFM images were analyzed with the software Gwyddion[1] after image processing such as horizontal scar correction and polynomial background removal. UV-Vis measurements were carried out with the Lambda 365 UV-Vis Spectrophotometer (PerkinElmer, USA). Thermogravimetric analysis by TGA 4000 Thermogravimetric Analyzer (PerkinElmer, USA) was chosen to assess the thermal degradation of various materials including UV-cured reference resin, dried TOCNFs powder and UV-cured nanocomposite film to compare their thermal stability with a temperature ramp from 30 to 600 °C at a rate of 10 °C/min in air. The flow behavior of monomers, as well as reference and nanocomposite resins were monitored by the rheometer MCR 302e (Anton-Paar, Austria) in plate-plate configuration at 1 mm distance with 1% strain and shear rates ranging between 0.1 and 100 s^{-1}. An AR 2000ex Rheometer (TA Instruments, USA) was used to perform photo-rheometry analysis of resins at 1% strain and 1 Hz frequency at room temperature. The resins were subjected to ~ 15 mW/cm^2 light intensity via a 200 W, high pressure Hg lamp (S2000 OmniCure UV3-Surface Cure Lamp, Exfo, Canada) calibrated with the Silver Line UV Radiometer (CON-TROL-CURE, Germany). Photo-differential scanning calorimetry (DSC Q100, TA Instruments, USA) analysis was carried out

isothermally on specimens by applying a UV light intensity of 0.5 mW/cm^2 via two optical fiber guides (as for the sample and the reference) with the OmniCure S2000 source) after 2 minutes of waiting for stabilization at room temperature (25 °C) in a nitrogen atmosphere. The peak of the exotherm, total reaction time and recorded heat flow were used to follow the advancement of the photo-polymerization reaction. The hardness and the reduced modulus data were acquired through nanoindentation measurements. Hysitron TI 950 TriboIndenter nanoindenter (Bruker,USA) was used to indent square arrays at 16 to 100 locations with each indentation separated by 15 µm distance. 10 to 150 µN force was applied and the unloading curves were fit with a power law to calculate the hardness and the reduced modulus of the tested specimen[2]. Wear tests were performed with a linear, reciprocating ball-on-disk nanotribometer, NTR3 (Anton-Paar, Austria), using 2 mm wide 100Cr6 steel balls (DIN 5401 – G40) as counterpart at a frequency of 1 and 5 Hz and normal forces ranging between 10 and 500 mN. The steel balls were cleaned with isopropanol before each test, which consisted in 5000 cycles on a 2 mm long track. To ensure the result reproducibility, 4 coatings with varying TOCNFs volume concentrations (0, 0.5, 5 and 10 vol.%) were measured 3 times using 10 and 20 mN loads at 1 Hz frequency as reported in Table 3. Following the wear tests, the track profiles were imaged with a laser scanning confocal microscope (VK-X250, Keyence, Japan) to quantify the wear volume. The water contact angle (WCA) of the flat and texturized coatings was measured using the DSA20E Easy Drop Goniometer (Krüss, Germany) with three or five repetitions and 2 or 8 µL droplets. As a complementary assessment, self-cleaning tests were performed on the nanoimprinted composite films by placing pepper grains on tilted samples and observing the sliding water droplets that can potentially remove the grains from the surface. Pepper grains were selected as model hydrophobic contaminants to prove the self-cleaning character of the imprinted surfaces. Furthermore, scanning electron microscopy (Gemini-SEM 300, Zeiss, USA) images of the coatings were acquired with In-Lens detector at a working distance of 3 mm and acceleration voltage of 1 kV, and the secondary electrons external detector (SE2) at a working distance of 6 mm and acceleration voltage of 3 kV. For preparation, a 10-nanometer thick layer of iridium was deposited on the samples with the Q150T magnetron sputtering multi-coater

(Quorum Technologies, UK). Lastly, the focused ion beam milling of the previously iridium-coated samples and subsequent imaging and elemental mapping were done with NVision 40 (Zeiss, Germany). Gallium cations (Ga^+) were used for milling of initially protective carbon-deposited surface structure, which is described as bump-on-bump in the main article. 4 kV acceleration voltage was utilized for both the imaging and the elemental mapping.

Pre-hydrolysis of surface modifiers

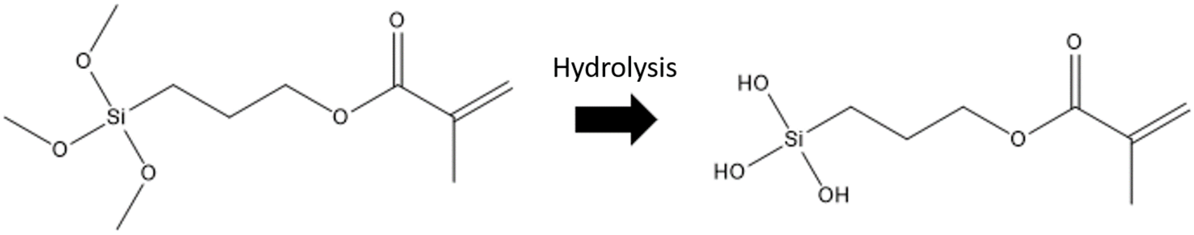

Figure S2. Schematic representation of the change in the molecular structure due to the hydrolysis step of MPS; specifically, CH_3 groups are replaced by OH groups.

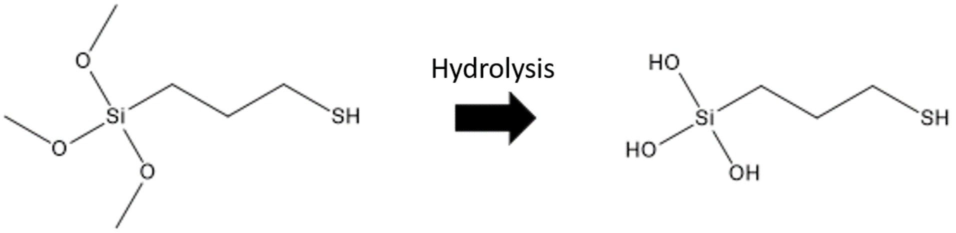

Figure S3. Schematic representation of the change in the molecular structure due to the hydrolysis step of MPTMS; specifically, CH_3 groups are replaced by OH groups.

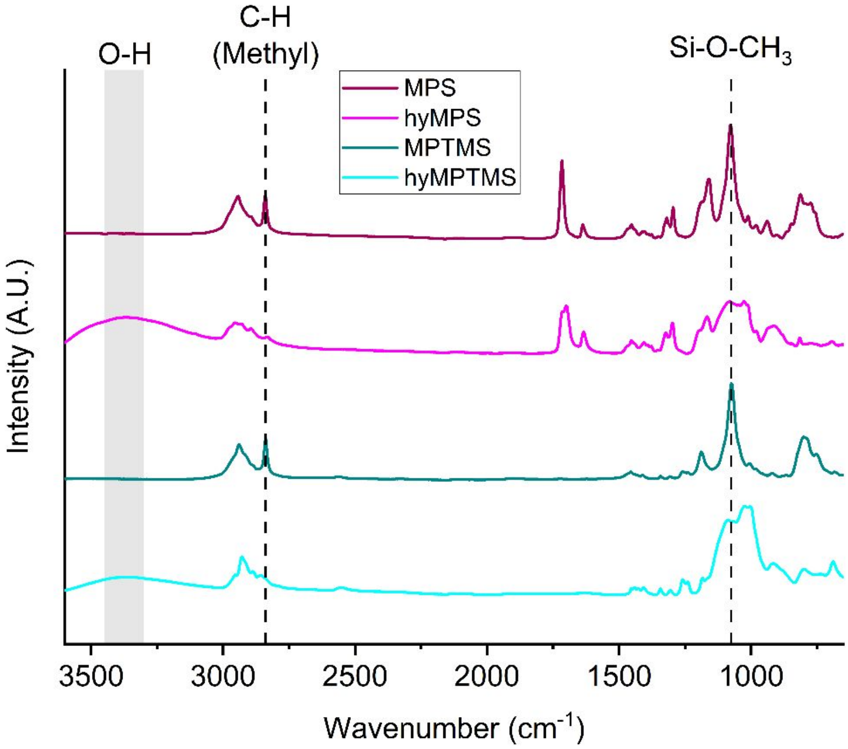

Figure S4. FT-IR spectra showing the pre-hydrolysis of MPS and MPTMS molecules.

The peaks of Si-O-CH_3 at 1075 cm^{-1} and the methyl C-H stretch at 2840 cm^{-1}, present in both MPS and MPTMS silanes, decreased following the pre-hydrolysis. In addition, the broad O-H stretch from the formed hydroxyl groups and adsorbed water are visible, indicated between 3300 and 3450 cm^{-1} for both pre-hydrolyzed silanes.

Variation in light transmittance based on filler concentration

The increase in filler volume percentage could decrease the transparency of the resin causing a white coloring as can be observed in Figure S5.

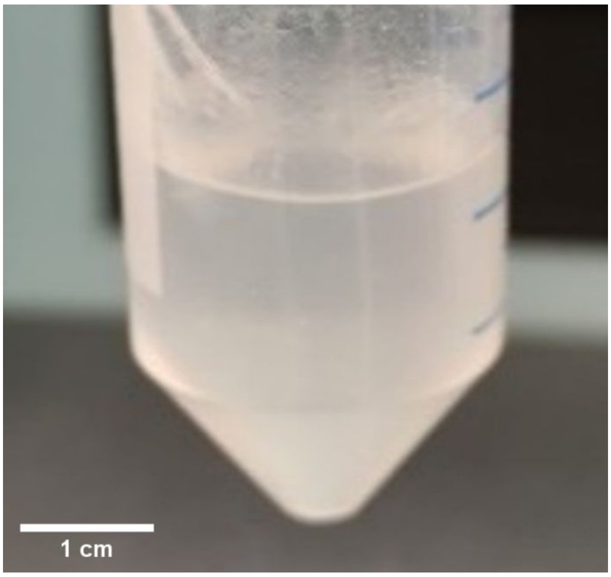

Figure S5. Image of the suspension of the double-modified TOCNFs (Bu_4NOH and MPS) in thiol-ene resin with 10 vol.% TOCNFs loading.

The cured nanocomposite films had highly differing surface roughness values as the concentration of fillers vary (Figure S6) with lowest roughness observed belonging to the sample with the lowest filler concentration.

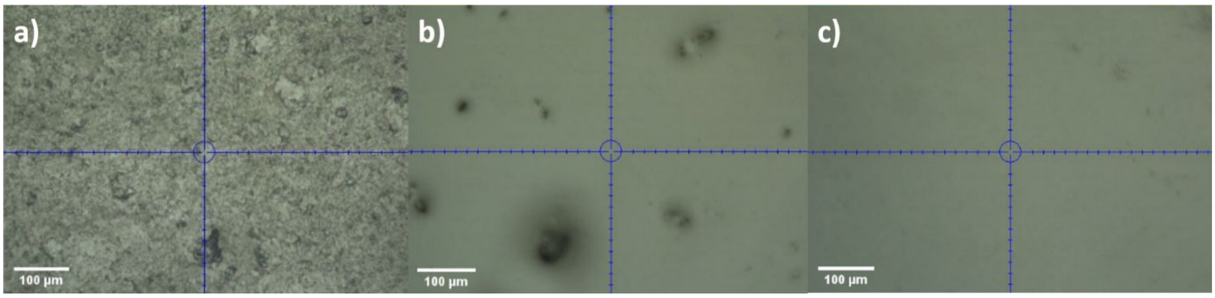

Figure S6. Optical microscopy images showing the surface roughness of cured nanocomposite films containing a) 10 vol.% Bu_4NOH-hyMPS-TOCNFs, b) 5 vol.% Bu_4NOH-hyMPS-TOCNFs and c) 1 vol.% Bu_4NOH-hyMPS-TOCNFs.

The low roughness contributed to the high transmittance of cured films on glass slides (Figure S7).

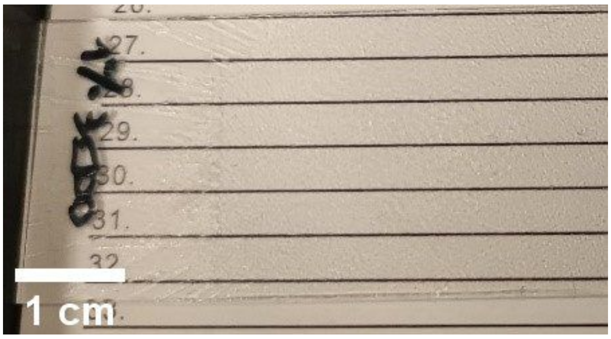

Figure S7. Transparent UV-cured nanocomposite film with 1 vol.% Bu_4NOH-hyMPS-TOCNFs on a glass slide.

Nanocomposite films with 85-90% transmittance within visible wavelength range (Figure S8), were prepared and measured by UV-Vis method. The high transmittance of these films could be considered similar to the cured reference resin above approx. 380 nm. If the concentration of TOCNFs was sufficiently low (in particular ≤ 1 vol.%) and surface modifications were applied, the formulation could lead to well-dispersed fillers inside the polymer matrix. The final transmittance of the films nevertheless also depended on the chosen processing method and its parameters which ultimately controlled the filler dispersion level and the cured film surface roughness. Thus, only a few selected UV-Vis graphs are displayed in Figure S8 to support the composite film transparency claims, which were obtained by doctor blading (~ 200 µm) prior to UV curing.

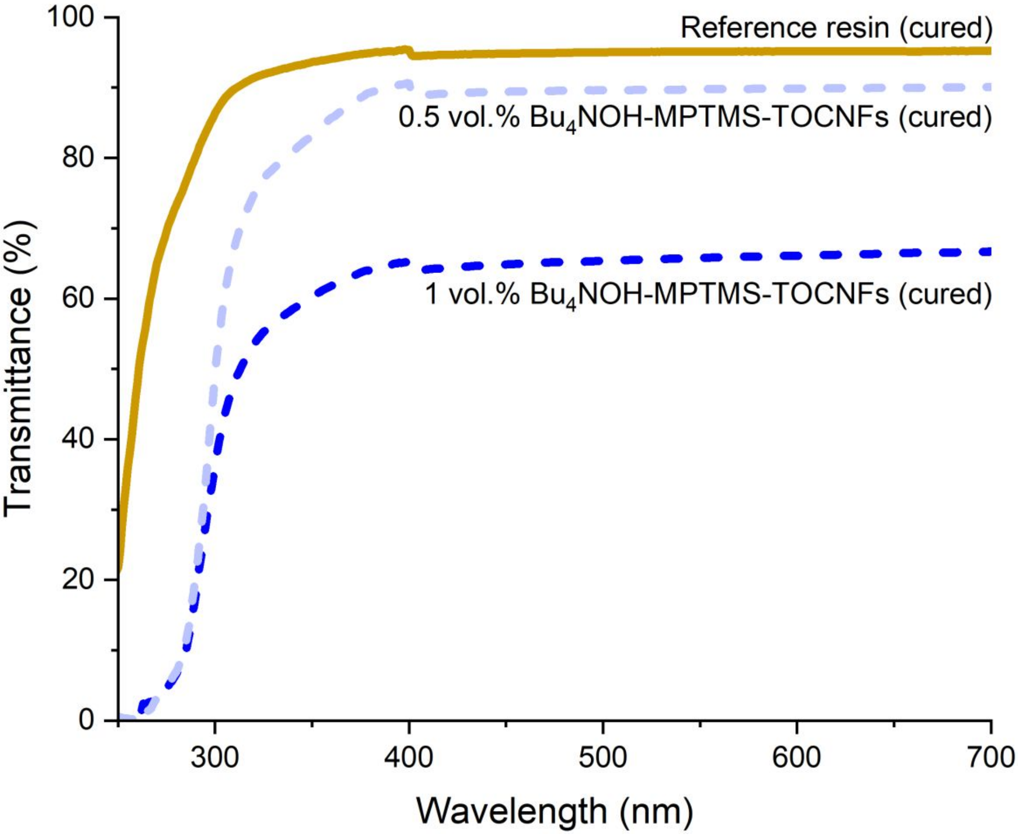

Figure S8. UV-Vis spectra of UV-cured nanocomposite films with different compositions compared to UV-cured reference resin film with visible-to-UV lamp switch occurring at 400 nm.

Improvement in particle dispersion following cation exchange

For further investigation regarding the Bu_4NOH surface modification of TOCNFs, zeta potential (Table S1) measurements were performed. The decrease in the modulus of the zeta potential following surface modification with Bu_4NOH indicates that the modified cellulose nanofibrils exhibited a lower repelling power and thus lower stability in the aqueous medium. In contrast, dispersion in a hydrophobic medium, such as the resin used in this project (Figure S5), has yielded a more transparent and viscous resin in agreement with the estimations based on zeta potential. However, the zeta potential value of the Bu_4NOH-TOCNFs water suspension may not have been quantitatively accurate as it had a basic pH, unlike the neutral water suspension with TOCNFs, which also had an influence on the zeta potential. Thus, the results could be evaluated only qualitatively.

Table S1. Obtained zeta-potentials concerning TOCNFs and Bu_4NOH-TOCNFs water suspensions (0.1 wt.%)

Particle type	Medium	Concentration [wt.%]	Zeta potential [mV]	Literature reference [mV]
TOCNF	water	0.1	-54.5	-55.8 [3]
Bu_4NOH-TOCNF	water	0.1	-39	-

Morphologies and sizes of modified TOCNFs

As received TOCNFs

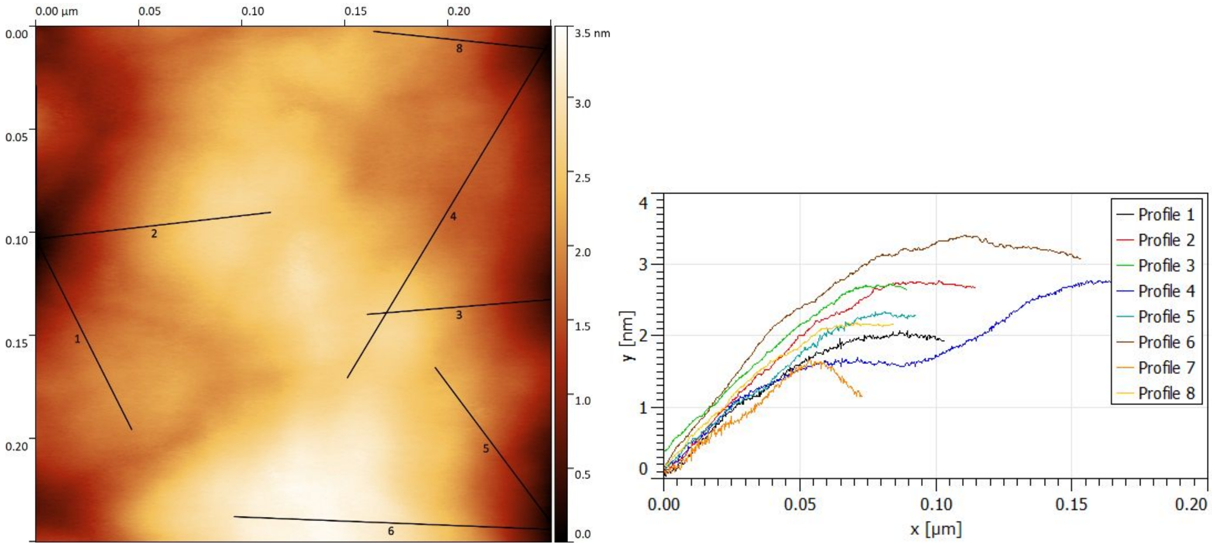

Figure S9. First AFM image of as received TOCNFs (left) and the obtained height profiles (right) with heights ranging between 1.2 – 3.2 nm and on average 2.1 ± 0.6 nm.

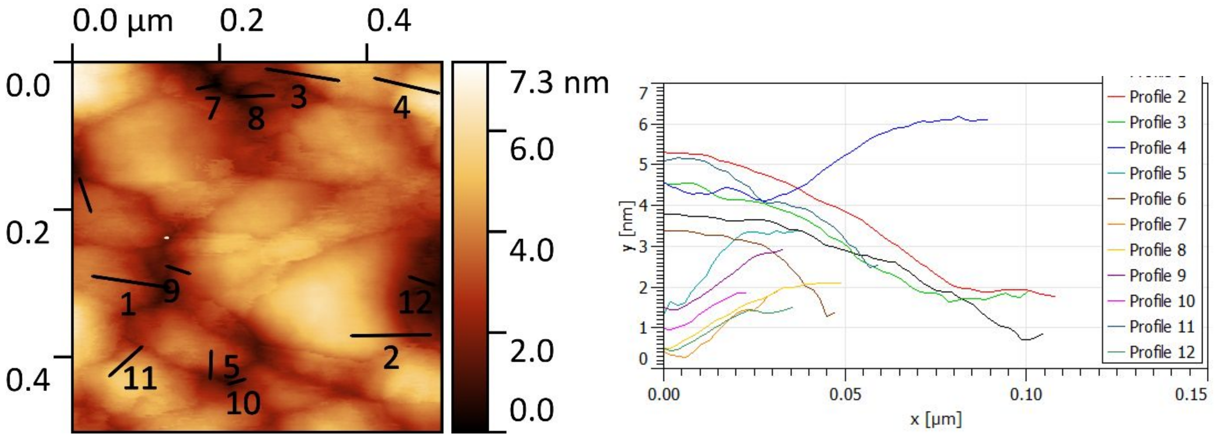

Figure S10. Second AFM image of as received TOCNFs (left) and the obtained height profiles (right) with heights ranging between 1.0 – 3.3 nm and on average 1.8 ± 0.7 nm.

Overall height range is 1.0 – 3.3 nm and the overall height average is 1.9 ± 0.6 nm.

Bu$_4$NOH-TOCNFs

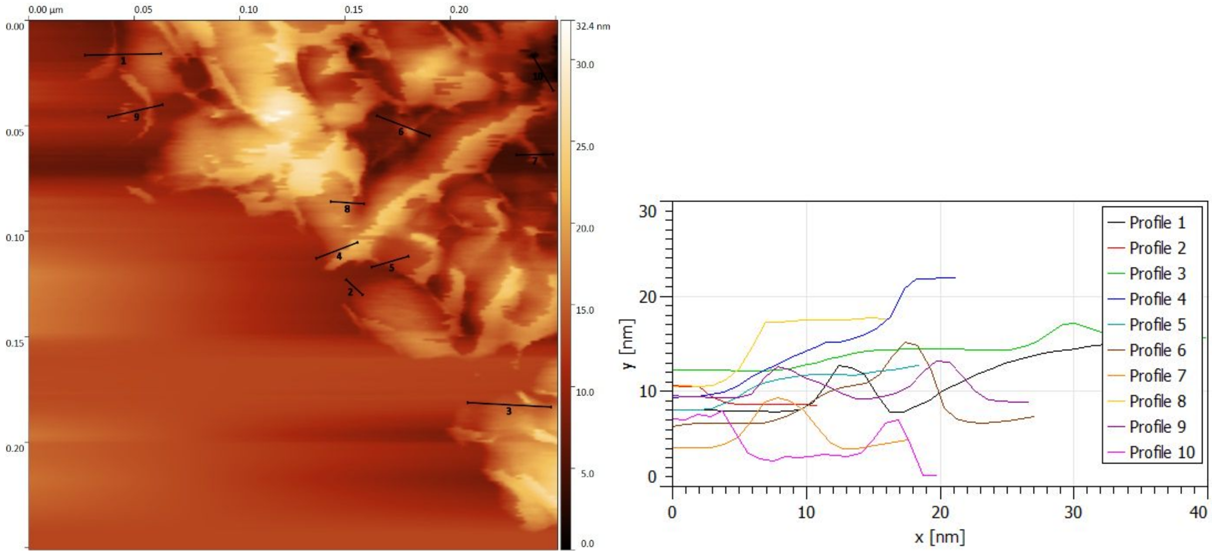

Figure S11. First AFM image of Bu$_4$NOH-TOCNFs (left) and the obtained height profiles (right) with heights ranging between 2.0 – 6.9 nm and on average 4.1 ± 1.5 nm.

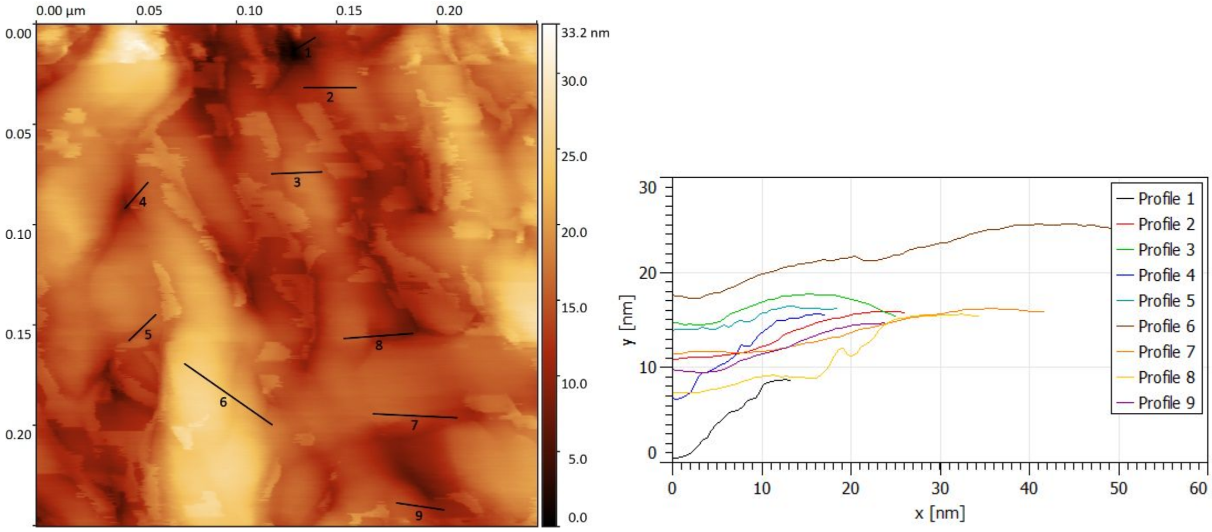

Figure S12. Second AFM image of Bu$_4$NOH-TOCNFs (left) and the obtained height profiles (right) with heights ranging between 1.4 – 4.6 nm and on average 2.9 ± 0.9 nm.

Overall height range is 1.4 – 6.9 nm and the overall height average is 3.5 ± 1.3 nm.

Bu$_4$NOH-hyMPS-TOCNFs

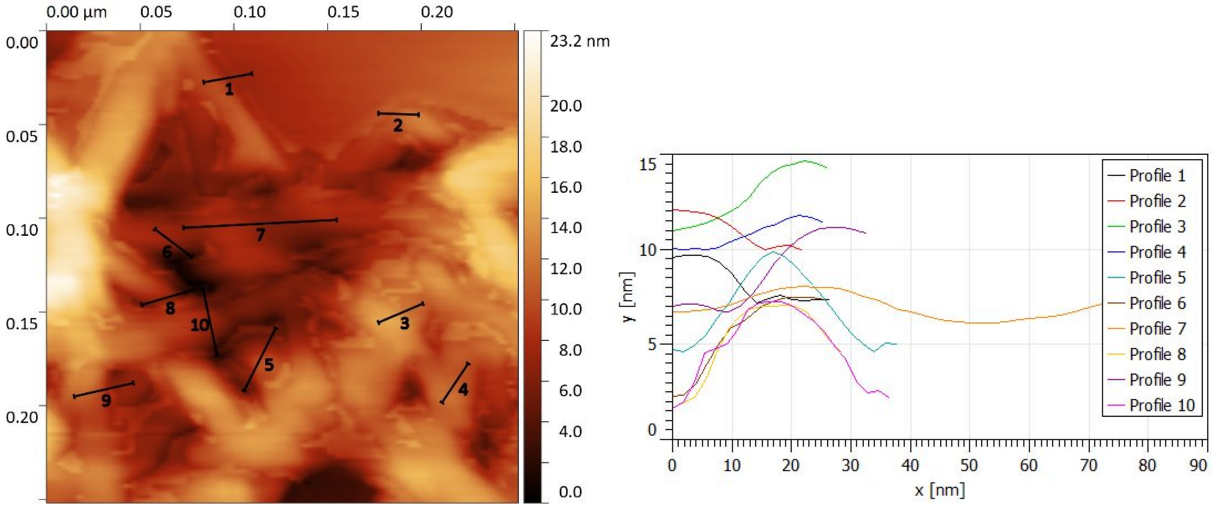

Figure S13. AFM image of Bu$_4$NOH-hyMPS-TOCNFs (left) and the obtained height profiles (right) with heights ranging between 1.8 – 5.7 nm and on average 3.9 ± 1.5 nm.

Bu$_4$NOH-hyMPTMS-TOCNFs

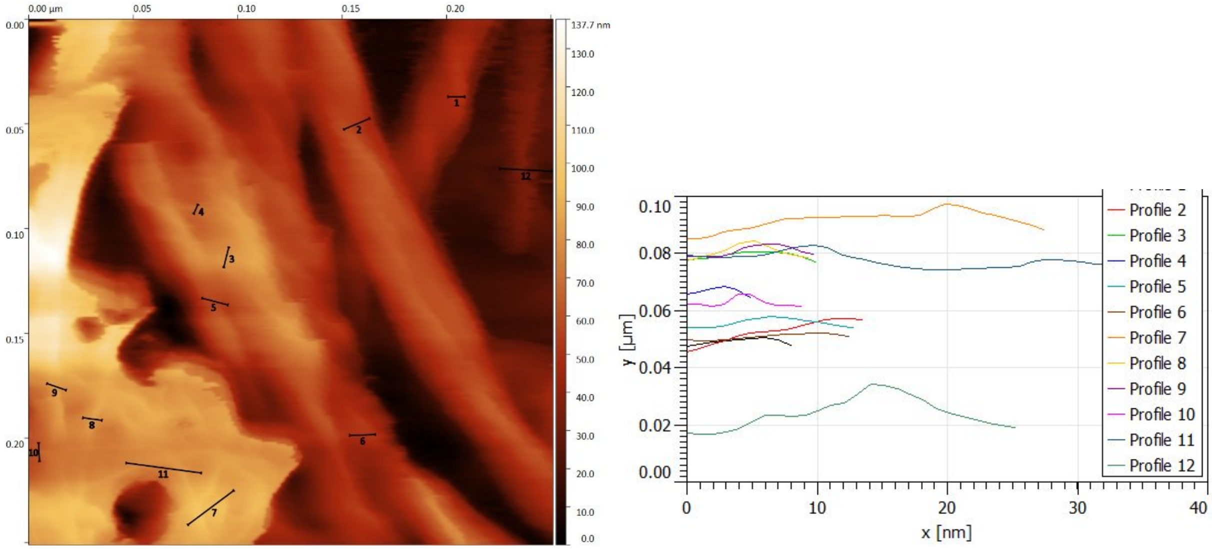

Figure S14. First AFM image of Bu$_4$NOH-hyMPTMS-TOCNFs (left) and the obtained height profiles (right) with heights ranging between 2.0 – 4.3 nm and on average 3.4 ± 0.8 nm.

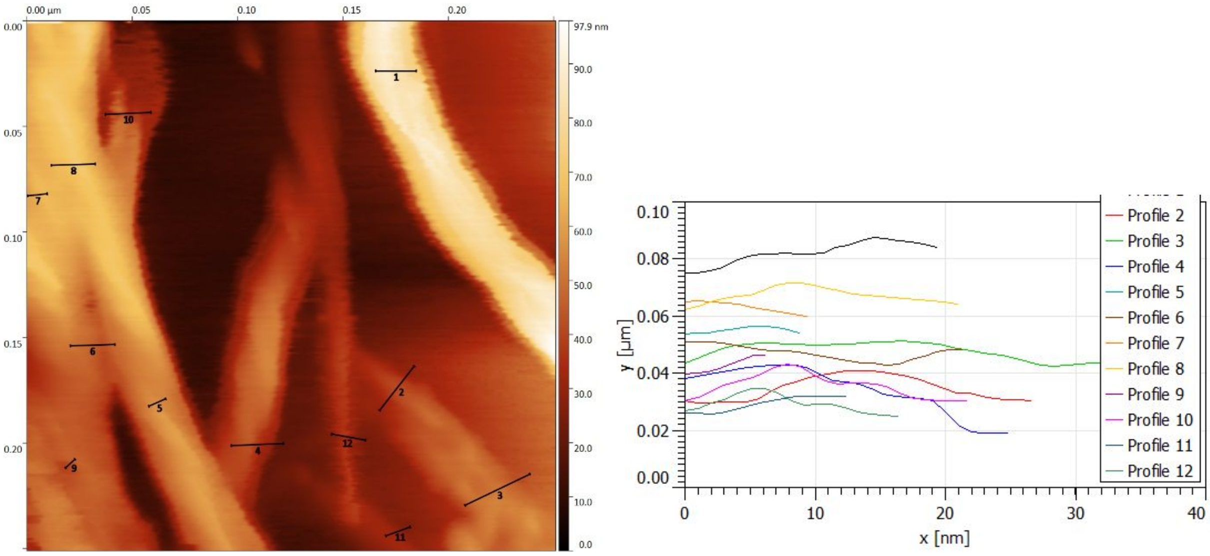

Figure S15. Second AFM image of Bu_4NOH-hyMPTMS-TOCNFs (left) and the obtained height profiles (right) with heights ranging between 2.0 – 7.5 nm and on average 4.3 ± 1.5 nm.

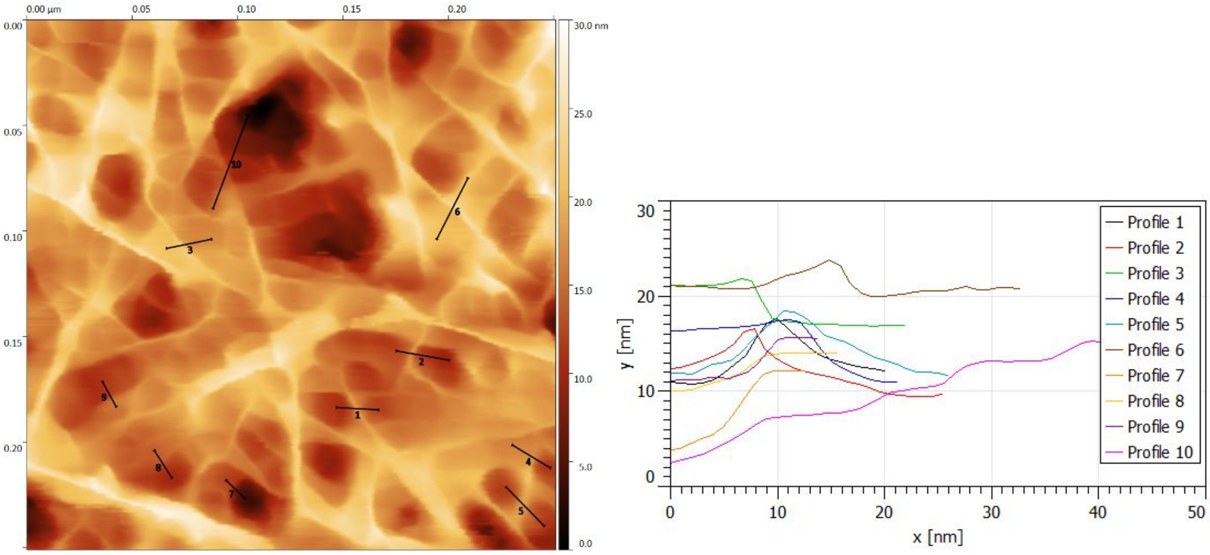

Figure S16. Third AFM image of Bu_4NOH-hyMPTMS-TOCNFs (left) and the obtained height profiles (right) with heights ranging between 2.1 – 8.1 nm and on average 4.8 ± 1.8 nm.

Overall height range is 2.0 – 8.1 nm and overall height average is 4.2 ± 1.5 nm.

Photopolymerization of thiol-ene reference resin

FT-IR analysis (Figure S17) was used to evaluate the thiol-ene photochemical reaction between TMPDE and TMPTMP, following the photoinitiation by the Norrish I type photoinitiator 2,2-dimethoxy-2-phenylacetophenone. By comparing the FT-IR spectra of the different chemical species, the disappearance of the C=C (920, 1645 and 3080 cm^{-1}) and S-H (2565 cm^{-1}) groups, which are solely present in monomers, was evident and confirmed the photo-cross-linking reaction.

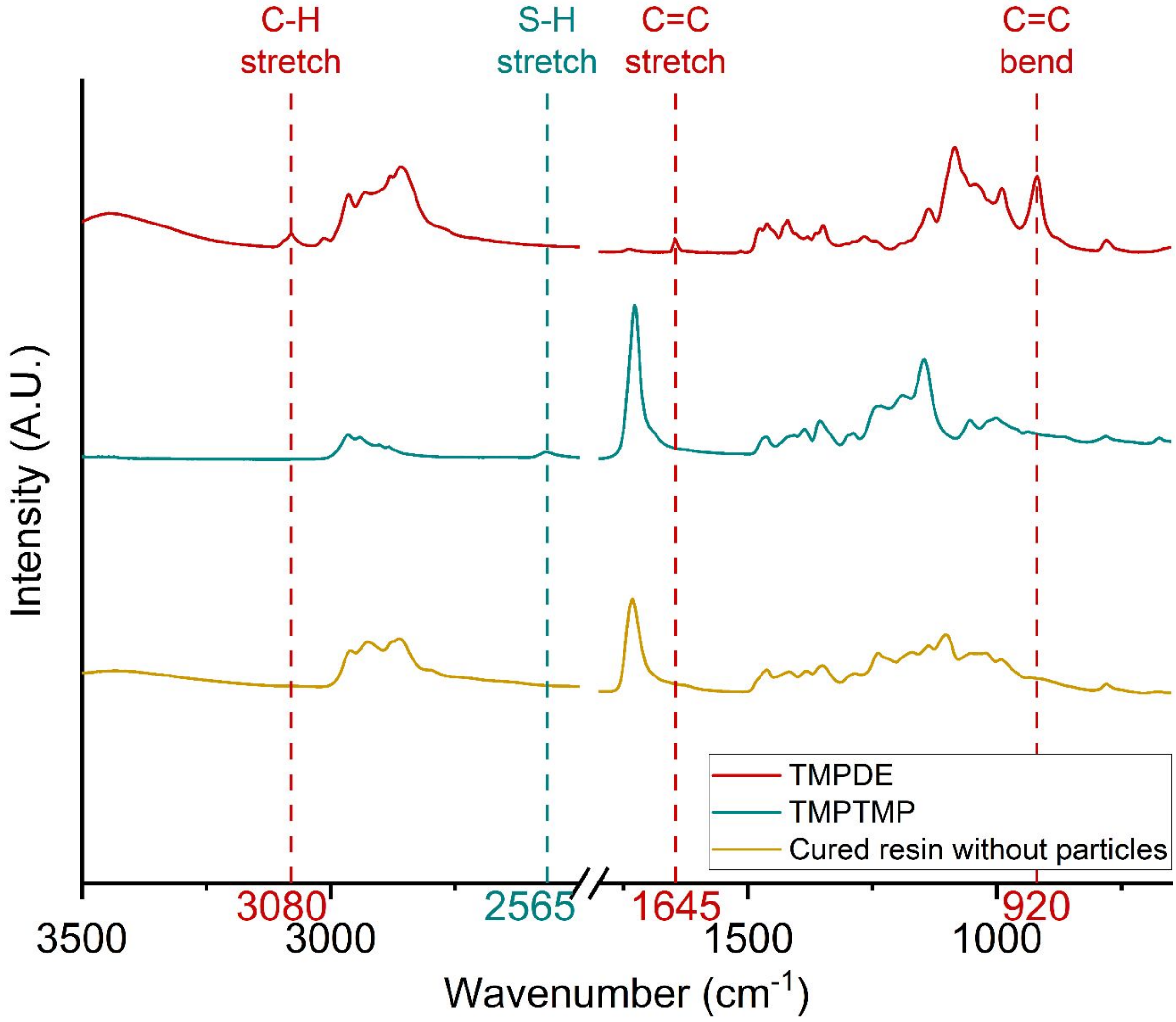

Figure S17. FT-IR spectra of TMPDE, TMPTMP and UV-cured reference resin.

Photo-curing of composite resins with changing viscosity and heat flow

Figure S18 displays the viscosity increase in resins during photo-curing with respect to the irradiation duration and includes also the control samples, which were separately prepared for repeatability. These samples showed negligible to minor deviations from the initial tests.

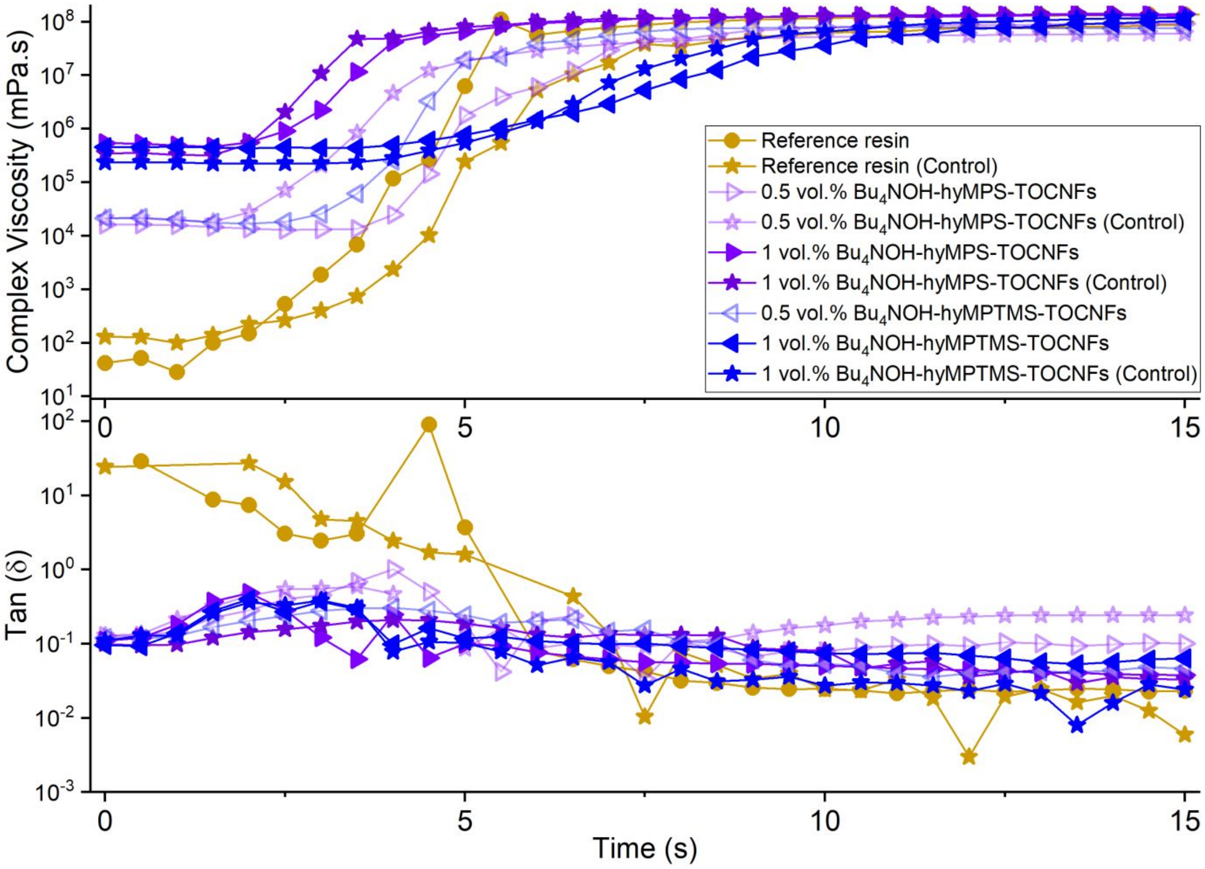

Figure S18. Photo-rheometry curves of reference and nanocomposite resins with different compositions, subjected to ~ 15 mW/cm^2 light intensity at time t = 0 s to analyze the photo-cross-linking kinetics.

In Figure S19, it can be seen that the photo-DSC measurements had a decent repeatability insofar as showing distinguishable trends between resins with varying filler concentration and surface modification type. Moreover, the composite resins with 0.5 vol.% MPTMS-grafted TOCNFs also showed reduced photo-curing speeds but to a lesser extent in comparison with resins having 1 vol.% of MPTMS-grafted TOCNFs.

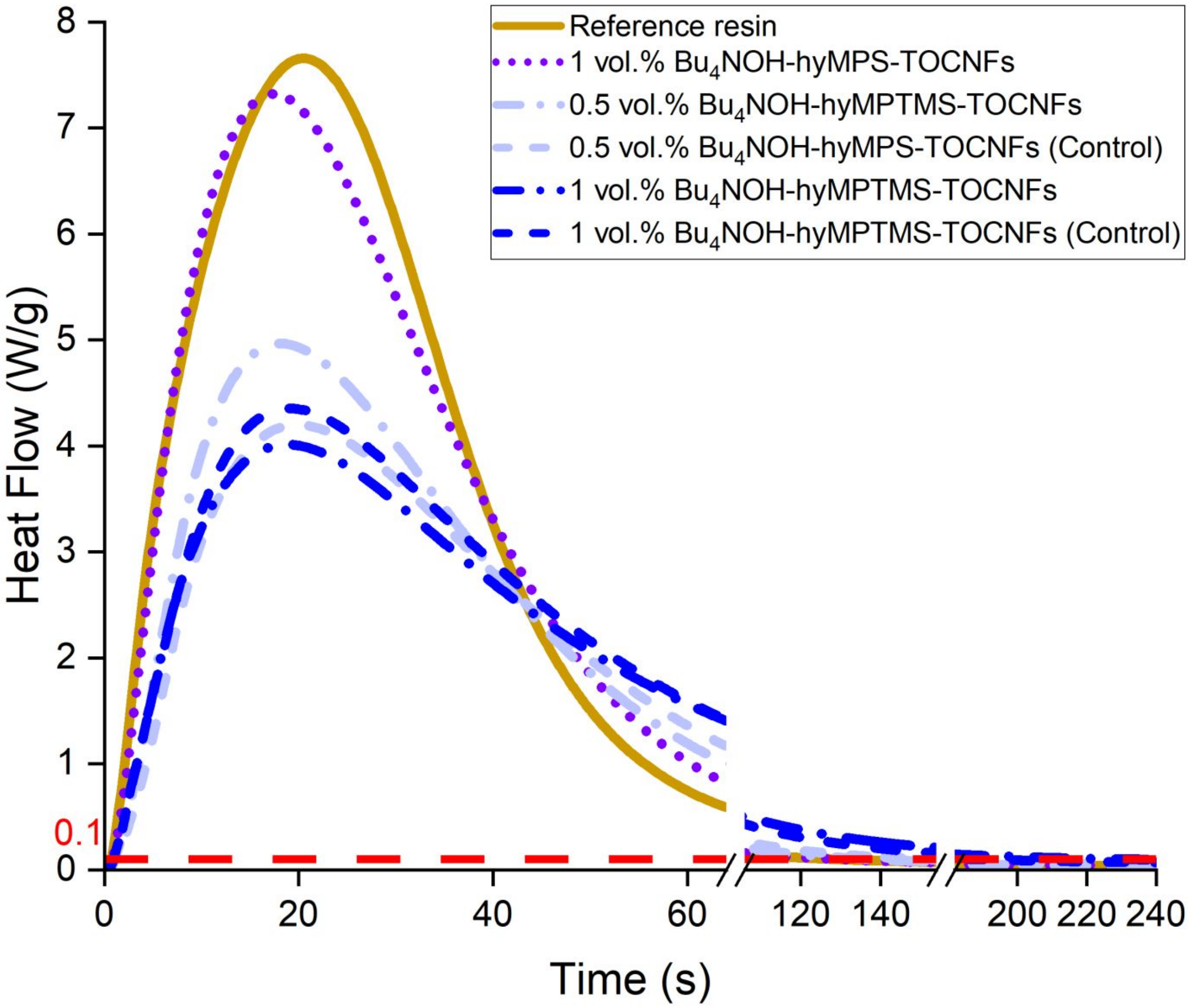

Figure S19. Photo-DSC measurements with additional curves demonstrating repeatability of resin curing behavior as well as the effect of reduced particle loading on reaction speed.

Thermal degradation of as received TOCNF

Dehydration, depolymerization, decomposition of glycosyl units and the residual char formation[4] are shown in Figure S20 with the main onsets of the weight graph given as dashed reference lines. The depolymerization involved the degradation of sodium anhydroglucuronate units (the decarboxylation step)[5] and two main depolymerization regions with DTG peaks at ~ 240 and ~ 290 °C. These peaks could be ascribed to the degradation of the oxidized (which begins with decarboxylation) and the original cellulose[5,6].

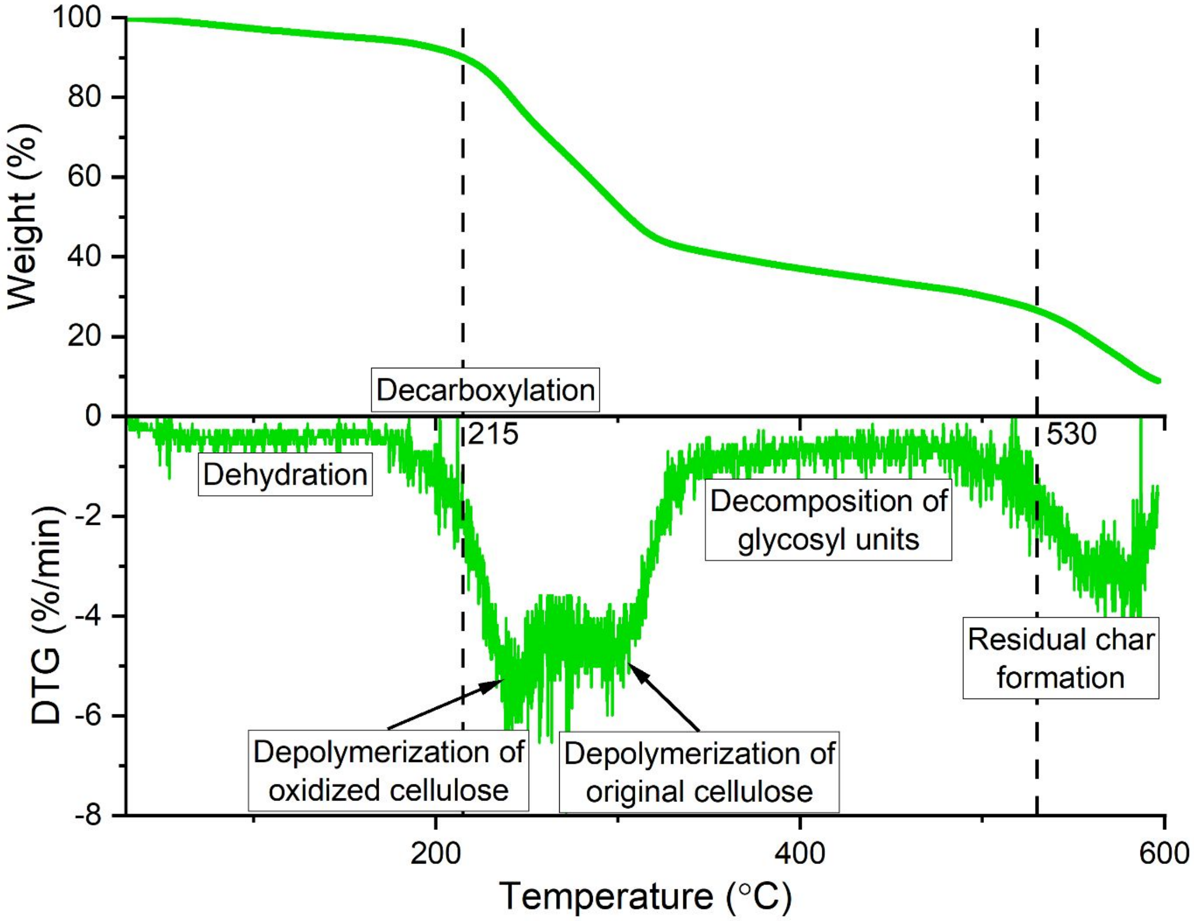

Figure S20. TGA and derivative TGA curves of dried TOCNF powder tested in air between 30 - 600 °C with a constant rate of 10 °C /min with the thermal degradation steps.

Tribological analysis of the composite coatings

The reference and 0.5 vol.% Bu_4NOH-hyMPS-TOCNFs loaded composite were tested 3 times for reproducibility with parameters 10, 20 mN and 1, 5 Hz. Figure S21 displays the two-step CoF reduction of the reference material with the second decrease occurring only after ~ 1000 cycles.

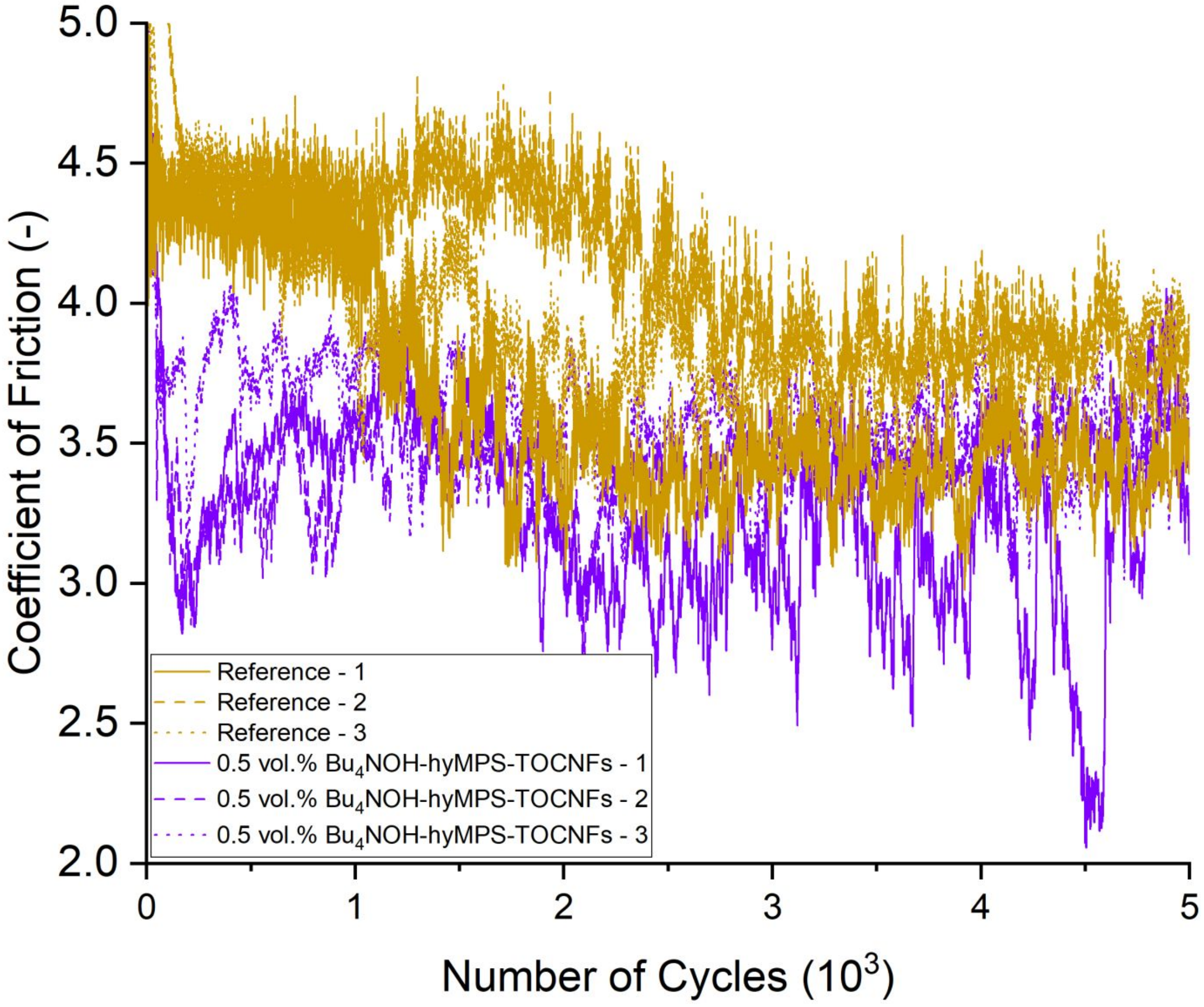

Figure S21. Coefficient of friction graphs of the reference material and the 0.5 vol.% Bu_4NOH-hyMPS-TOCNFs loaded composite with 10mN normal force and 1 Hz frequency.

Figure S22 shows the coefficient of friction and wear volume loss of the reference coating together with the 0.5 vol.% Bu_4NOH-hyMPTMS-TOCNFs loaded composite obtained after 5000 cycles of oscillatory wearing against a steel ball.

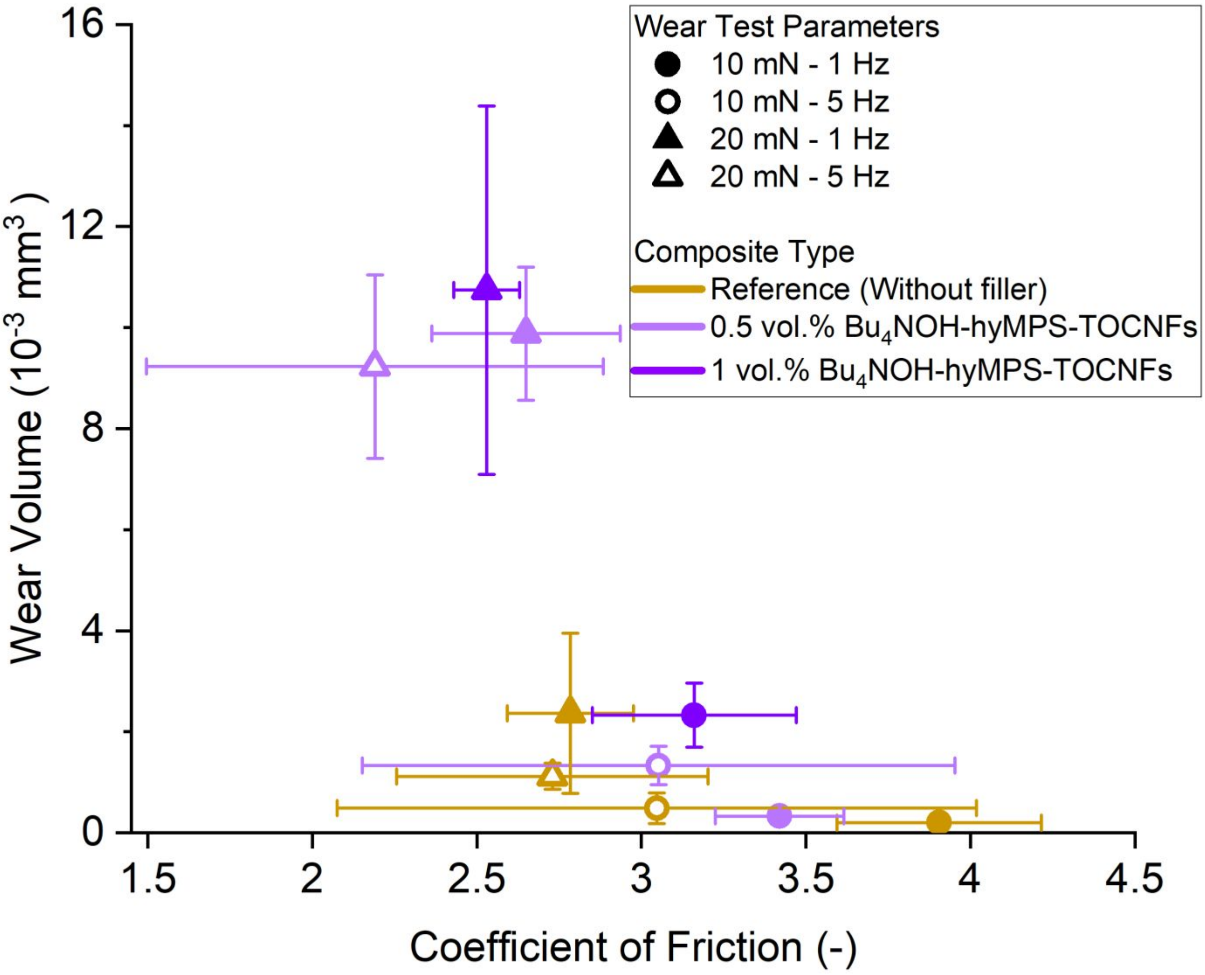

Figure S22. Wear volume against the coefficient of friction of the reference material against the 0.5 vol.% Bu_4NOH-hyMPS-TOCNFs loaded composite.

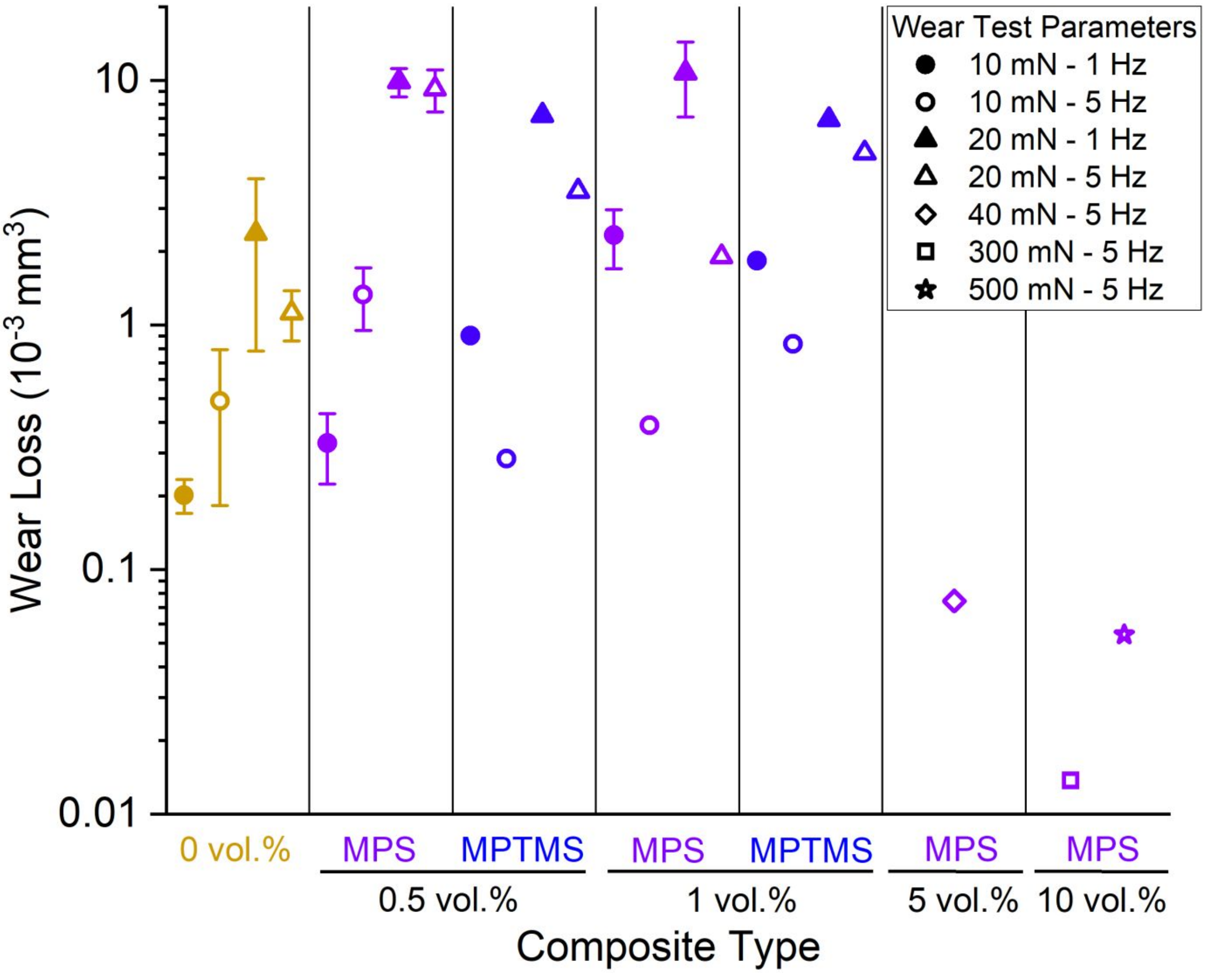

Figure S23. Wear loss results with changing parameters of material type, concentration, normal force and frequencies. Error bars are only given for the tests that were performed three times for repeatability.

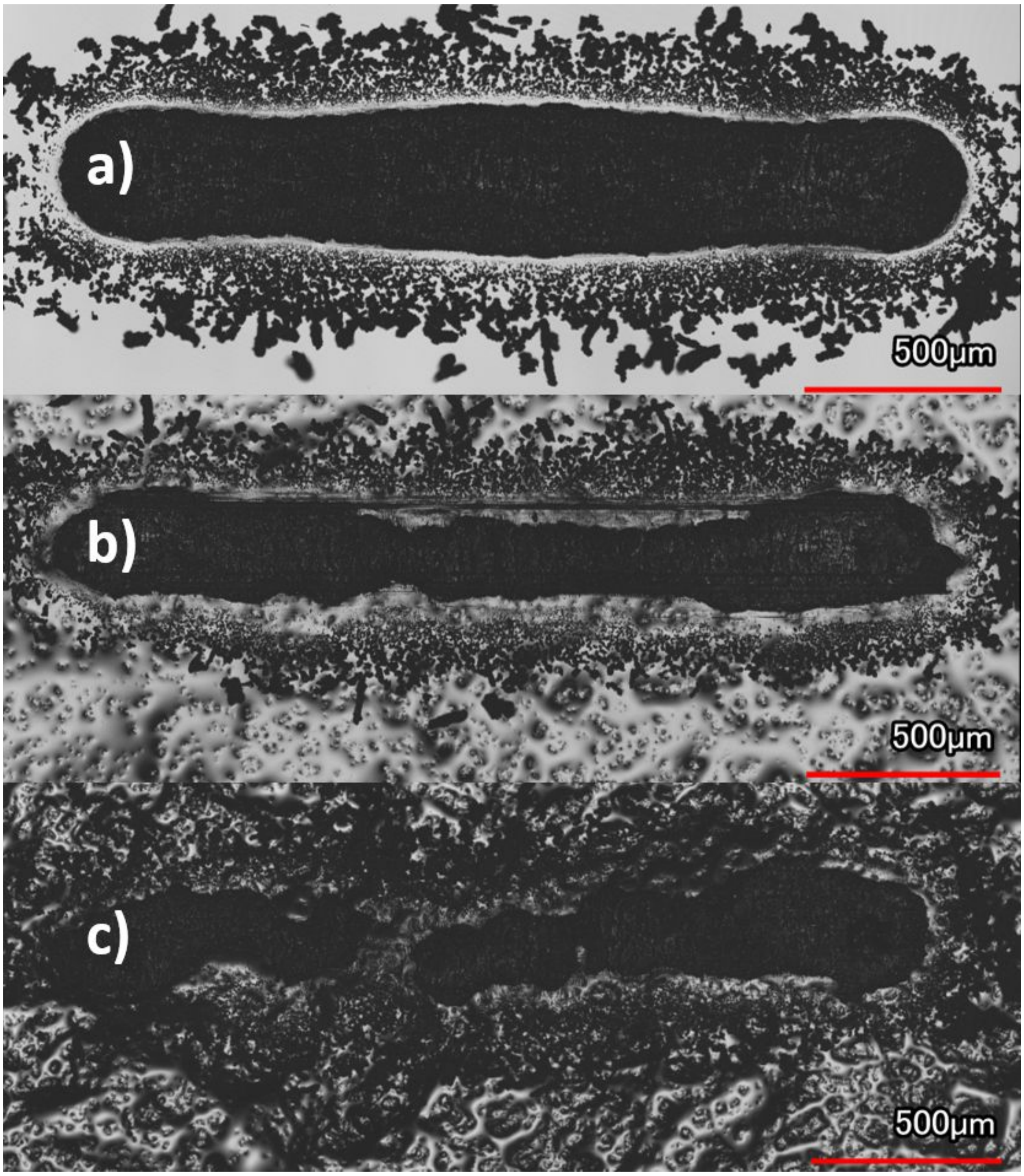

Figure S24. 2D confocal microscopy images showing 2 mm wear tracks of a) 0, b) 0.5, c) 1 vol.% Bu_4NOH-hyMPS-TOCNFs loaded composites under 10 mN force and at 1 Hz frequency.

Contaminated UVNIL pattern template surface following polymer film peel off

As shown in the SEM image (Figure S25), a portion of the cured film remained attached to the PDMS mold leading to an imperfect replication and rendering the mold incapable for repeated use.

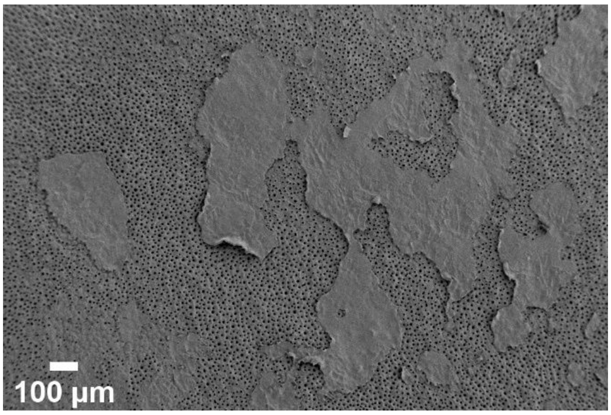

Figure S25. SEM image of a PDMS mold surface after UVNIL imprinting of 1 vol.% Bu_4NOH-hyMPS-TOCNFs nanocomposite resin showing residues of the imprinted cured film following the polymer film peel off.

References

(1) Nečas, D.; Klapetek, P. Gwyddion: An Open-Source Software for SPM Data Analysis. *Open Physics* **2012**, *10* (1), 181–188. https://doi.org/10.2478/s11534-011-0096-2.
(2) Oliver, W. C.; Pharr, G. M. An Improved Technique for Determining Hardness and Elastic Modulus Using Load and Displacement Sensing Indentation Experiments. *Journal of Materials Research* **1992**, *7* (6), 1564–1583. https://doi.org/10.1557/JMR.1992.1564.
(3) Wei, J.; Chen, Y.; Liu, H.; Du, C.; Yu, H.; Zhou, Z. Thermo-Responsive and Compression Properties of TEMPO-Oxidized Cellulose Nanofiber-Modified PNIPAm Hydrogels. *Carbohydrate Polymers* **2016**, *147*, 201–207. https://doi.org/10.1016/j.carbpol.2016.04.015.
(4) Kumar, A.; Negi, Y. S.; Choudhary, V.; Bhardwaj, N. K. Characterization of Cellulose Nanocrystals Produced by Acid-Hydrolysis from Sugarcane Bagasse as Agro-Waste. *Journal of Materials Physics and Chemistry* **2014**, *2* (1), 1–8. https://doi.org/10.12691/jmpc-2-1-1.
(5) Fukuzumi, H.; Saito, T.; Okita, Y.; Isogai, A. Thermal Stabilization of TEMPO-Oxidized Cellulose. *Polymer Degradation and Stability* **2010**, *95* (9), 1502–1508. https://doi.org/10.1016/j.polymdegradstab.2010.06.015.
(6) Jonasson, S.; Bünder, A.; Niittylä, T.; Oksman, K. Isolation and Characterization of Cellulose Nanofibers from Aspen Wood Using Derivatizing and Non-Derivatizing Pretreatments. *Cellulose* **2020**, *27* (1), 185–203. https://doi.org/10.1007/s10570-019-02754-w.
